# Empowering biologists with multi-omics data: colorectal cancer as a paradigm

**DOI:** 10.1093/bioinformatics/btu834

**Published:** 2014-12-18

**Authors:** Jing Zhu, Zhiao Shi, Jing Wang, Bing Zhang

**Affiliations:** ^1^Department of Biomedical Informatics, ^2^Advanced Computing Center for Research and Education, ^3^Department of Electrical Engineering and Computer Science and ^4^Department of Cancer Biology, Vanderbilt University, Nashville, Tennessee, USA

## Abstract

**Motivation:** Recent completion of the global proteomic characterization of The Cancer Genome Atlas (TCGA) colorectal cancer (CRC) cohort resulted in the first tumor dataset with complete molecular measurements at DNA, RNA and protein levels. Using CRC as a paradigm, we describe the application of the NetGestalt framework to provide easy access and interpretation of multi-omics data.

**Results:** The NetGestalt CRC portal includes genomic, epigenomic, transcriptomic, proteomic and clinical data for the TCGA CRC cohort, data from other CRC tumor cohorts and cell lines, and existing knowledge on pathways and networks, giving a total of more than 17 million data points. The portal provides features for data query, upload, visualization and integration. These features can be flexibly combined to serve various needs of the users, maximizing the synergy among omics data, human visualization and quantitative analysis. Using three case studies, we demonstrate that the portal not only provides user-friendly data query and visualization but also enables efficient data integration within a single omics data type, across multiple omics data types, and over biological networks.

**Availability and implementation:** The NetGestalt CRC portal can be freely accessed at http://www.netgestalt.org.

**Contact:** bing.zhang@vanderbilt.edu

**Supplementary Information:** Supplementary data are available at *Bioinformatics* online.

## 1 Introduction

Technology advancements have enabled comprehensive characterization of genomic, epigenomic, transcriptomic, proteomic and metabolomic changes in tumor specimens. A prime example is the TCGA project’s generation of multiple types of omics data from hundreds of human tumor specimens for each of the more than 20 selected tumor types.

Bridging the gap between data generation and investigators’ ability to retrieve and interpret the data is essential to fully realize the biological and clinical value of the vast amount of omics data. To address this challenge, powerful but user-friendly data query systems such as the cBioPortal for cancer genomics (Cerami *et al*., 2012) have been established. Moreover, the UCSC Cancer Genomics Browser (Zhu *et al*., 2009) enables simultaneous visualization of various types of omics data within the context of genomic sequence, providing an excellent platform for integrating genome-anchored information.

Biological networks provide excellent functional contexts for exploring omics data; however, node-link diagram-based network visualization becomes inadequate as network size and data complexity increase (Gehlenborg *et al*., 2010). Using CircleMaps, the UCSC Interaction Browser allows simultaneous visualization of multiple omics datasets within the context of gene interactions (Wong *et al*., 2013). Although very useful, this approach cannot scale up to large networks. Previously, we developed NetGestalt (Shi *et al*., 2013) that exploits the inherent hierarchical modular architecture of biological networks to achieve high scalability. NetGestalt orders the nodes of a network along the horizontal dimension of a webpage based on the underlying hierarchical organization of the network. Visualization in the horizontal dimension conveys the functional relationship between different nodes (i.e. genes) as encoded in the network. The linear layout facilitates scaling up to thousands of genes. Moreover, because it only uses one dimension to display network nodes, node-related information from different data sources can be rendered as ‘tracks’ along the vertical dimension for visual comparison and integration. This unique approach makes NetGestalt an appropriate framework for the study of multidimensional cancer omics data in the context of biological networks.

Recent completion of the global proteomic characterization of the TCGA CRC cohort (TCGA Research Network, 2012) resulted in the first tumor dataset with complete molecular measurements at DNA, RNA and protein levels (Zhang *et al*., 2014). In this article, we describe the application of the NetGestalt framework, using CRC as a paradigm, to provide a network-centric view of multidimensional cancer omics data. We use three case studies to demonstrate that the portal not only provides for easy data querying and visualization but also enables efficient data integration within a single omics data type, across multiple omics data types and over biological networks.

## 2 Methods

### 2.1 Data architecture

**Fig. 1.**
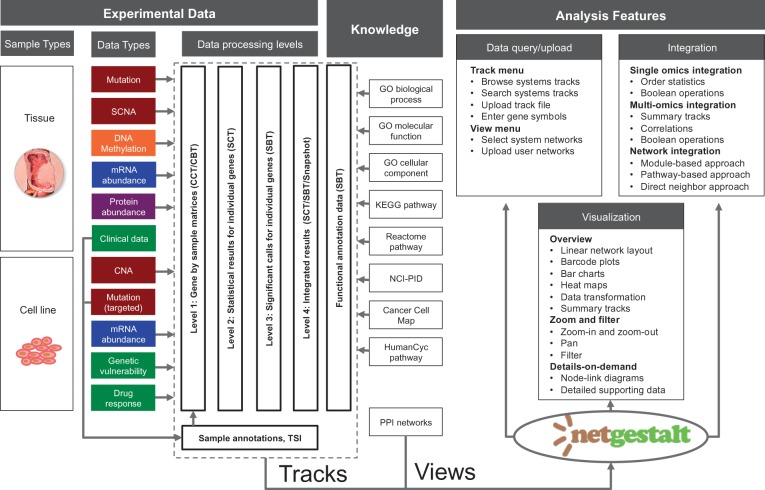
Data and analysis features in the NetGestalt CRC portal. The portal contains both experimental data and existing knowledge on pathways and networks. Experimental data are organized based on sample types, data types, and data processing levels. In the portal, networks provide functional **‘**views**’**, whereas all other data are converted to **‘**tracks**’**. The portal allows users to perform data query/upload, visualization and integrative analysis

As shown in Figure 1, experimental data in the portal are organized on the basis of sample types, data types and data processing levels. The portal also includes existing knowledge on pathways and networks. Details on data sources and data processing are provided in Supplementary Methods.

#### 2.1.1 Sample types

The portal includes data for both CRC tumors and cell lines. The former were collected from TCGA and the Gene Expression Omnibus (GEO), whereas the latter were from the Cancer Cell Line Encyclopedia (CCLE) project (Barretina *et al*., 2012) and the Achilles project (Cheung *et al*., 2011).

#### 2.1.2 Data types

For the TCGA tumor cohort, we collected (i) somatic mutation data for 224 tumor samples; (ii) somatic copy number alteration (SCNA) data for 257 tumor samples; (iii) DNA methylation data for 234 tumor samples and 42 normal samples; (iv) mRNA expression data for 222 tumor samples and 22 normal samples; (v) protein expression data for 90 tumor samples and 30 normal samples; and (vi) all available clinical data. Because patient survival data for the TCGA cohort are very limited, we further collected microarray gene expression data from GEO for five CRC cohorts. These cohorts have a total of 431 samples, with overall survival (OS) and disease-free survival (DFS) information for 326 and 318 samples, respectively. For cell lines from the CCLE project, we included (i) mRNA expression data for 61 CRC cell lines; (ii) copy number data for 58 CRC cell lines; (iii) mutation data for 61 CRC cell lines; and (iv) drug response data for 23 CRC cell lines. From the Achilles project, we further collected genes showing enhanced dependencies in the 18 CRC cell lines based on short hairpin RNA screens of 11 194 genes in 102 cell lines.

#### 2.1.3 Processing levels

Experimental data downloaded from various sources were processed at four different levels. At Level 1, data for individual samples were summarized at the gene level to generate gene-by-sample matrices. At level 2, for selected sample attributes, appropriate statistical analyses such as the Student’s *t*-test, Wilcoxon rank-sum test, Cox regression or Spearman’s correlation test were performed based on the matrices to generate test statistics, nominal *P* values, and False Discovery Rates (FDRs) for individual genes. At Level 3, based on pre-defined thresholds, such as FDR   <0.01 and/or fold change >2, significant genes were identified. At Level 4, data integration was performed. As an example, survival analysis results for the five GEO CRC cohorts were integrated to identify genes consistently correlated with survival time in multiple cohorts. As another example, correlations between SCNA and protein levels measured in the same tumor cohort were computed for individual genes to identify SCNAs that potentially drive protein abundance changes.

#### 2.1.4 Knowledge

The portal includes three protein–protein interaction networks: HPRD is based on the Human Protein Reference Database (Keshava Prasad *et al*., 2009), iRef is based on the iRef database (Turner *et al*., 2010) and iRef_HI expands the iRef network by further integrating ∼14000 interactions recently made available through an unbiased human interactome project (http://interactome.dfci.harvard.edu/H_sapiens/). We also included pathway data from Cancer Cell Map (10 pathways), GO Biological Process (808 GO terms), GO Cellular Component (231 GO terms), GO Molecular Function (383 GO terms), HumanCyc (267 pathways), KEGG (200 pathways), NCI Pathway Interaction Database (PID, 223 pathways) and Reactome (1108 pathways).

#### 2.1.5 File formats

All data described earlier were processed to one of the standardized data formats and stored on the web server. NetGestalt supports the *nsm* format for serialized and modularized networks, the *cct* format for composite continuous tracks (e.g. expression matrices), the *cbt* format for composite binary tracks (e.g. mutation matrices), the *sct* format for single continuous tracks (e.g. fold changes), and the *sbt* format for single binary tracks (e.g. significant genes). For composite tracks containing multiple samples, sample annotations can be stored in the *tsi* format. Detailed description of the file formats can be found in the Supplementary Manual. As shown in Figure 1, networks provide functional ‘views’ in NetGestalt, whereas all other data are converted to ‘tracks’, which can be queried, visualized and integrated using the analysis features in NetGestalt.

### 2.2 Data query and upload features

All data in the portal can be accessed through the menu bar located at the top of the web page. Under the ‘View’ menu, users can select one of the three network views (HPRD, iRef or iRef_HI) as the basis for data visualization and analysis. User-specific networks can also be uploaded through this menu. Under the ‘Track’ menu, users can browse all 3356 system tracks or search for a specific system track. All tracks in the current version of NetGestalt are summarized in Supplementary Table S1. Users can also upload their own track files in one of the NetGestalt file format or simply enter a list of gene symbols to create a new track.

### 2.3 Visualization features

Visualization in the portal follows the basic visual design principles summarized as the Visual Information Seeking Mantra (Shneiderman, 1996): Overview first, zoom and filter, then details-on-demand (Fig. 1).

#### 2.3.1 Overview

Genes in a network are ordered in one dimension based on the underlying hierarchical organization of the network, making it possible to provide an overview of data for all genes in a network, with composite tracks visualized as heat maps, single continuous tracks as bar charts, and single binary tracks as barcode plots. For a composite track, samples annotations can be co-visualized as a companion heat map, which also enables interactive sorting of the samples based on annotations of interest. For composite continuous and single continuous tracks, visualization may be improved through data transformation.

**Fig. 2.**
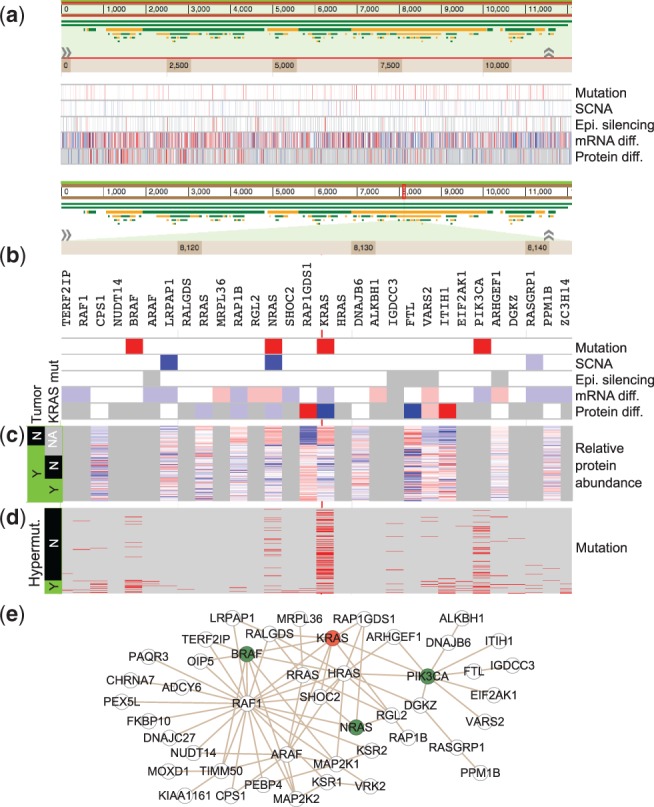
Retrieving information for *KRAS*. (**a**) The Omics snapshot summarizes the tumor-related somatic mutations, SCNAs, epigenetic alterations and differential expression at mRNA and protein levels for all genes. For somatic mutation, red represents significantly mutated genes and light red represents genes mutated in at least 5% of the CRC samples; for SCNA, red/light red represent genes in the focal amplification regions and blue/light blue represent genes in the focal deletion regions, with red/blue representing candidates drivers; for epigenetic alteration, red represents epigenetically silenced genes; for mRNA/protein expression, red and blue represent significantly over- and under-expressed genes with fold change ** >**2, whereas light red and light blue represent significantly over- and under-expressed genes with fold change ** <**2. Grey represents missing data. (**b**) When zooming into the *KRAS* gene, it can be seen that *KRAS* is significantly mutated in CRC and down regulated at both mRNA and protein levels in tumors compared with normal colon biopsies. Data for genes in the network neighborhood are visualized simultaneously. (**c**) The proteomics data show that the *KRAS* protein abundance is lower in tumors compared with normal controls; however, tumors with mutant *KRAS* have higher *KRAS* abundance compared with those without mutant *KRAS*. Red and blue represent relative over- and under-expression, respectively. (**d**) Sample-level mutation information for *KRAS* and genes in this network neighborhood. (**e**) Protein**–**protein interactions between *KRAS* and genes in this network neighborhood

To provide a concise overview for the multidimensional omics data for the TCGA tumor cohort, we created an ‘omics snapshot’ track that summarizes all tumor-related molecular alterations in five sub-tracks corresponding to somatic mutations, SCNAs, epigenetic alterations, and differential expression at mRNA and protein levels, respectively (Fig. 2a). Similarly, a ‘clinical relevance snapshot’ track was created with four sub-tracks indicating markers for DFS, markers for OS, signature genes for the microsatellite instable (MSI) versus microsatellite stable (MSS) comparison, and signature genes for the stage IV versus stage I comparison, respectively. Each sub-track was generated by meta-analysis of multiple independent studies when possible. Based on the cell line data, a ‘drug response snapshot’ track was generated with 24 sub-tracks each representing the correlations between the sensitivity of the 23 cell lines to one of the 24 drugs and mRNA expression of individual genes across the cell lines.

#### 2.3.2 Zoom and filter

Because the one-dimensional layout of network nodes is created on the basis of the hierarchical modular organization of the network, a quick overview of one or multiple data tracks can often reveal network modules or sub-networks with interesting patterns, e.g. modules enriched with mutated or differentially expressed genes. Accordingly, users can easily zoom into the areas of interest. When a pattern cannot be easily recognized by visual examination, network analysis methods described in the ‘Integrative analysis features’ section (see below) can be used. If a single gene of interest is pre-specified, it is also possible to directly zoom to the gene. When zoomed-in, users can also pan to left or right and adjust the zoom level to explore the data.

Although the network-derived 1D layout of the genes provides a convenient functional framework for data visualization, many important functional relationships between genes cannot be revealed in such a layout due to the complexity of biological systems and the multi-functional nature of the genes. We address this limitation by the filtering feature associated with single binary tracks, through which a subset of data for the genes in a single binary track can be retrieved and visualized. In NetGestalt, single binary tracks can be defined based on experimental data (e.g. recurrently mutated genes or genes significantly correlated with survival time), existing knowledge on pathways, or user provided gene lists. Moreover, through the filtering feature associated with single continuous tracks, it is also possible to apply user-defined criteria to derive single binary tracks.

#### 2.3.3 Details-on-demand

For all data tracks, after zooming into a network area with less than a pre-specified number of genes (i.e. 500 in the current implementation), a node-link diagram can be used to visualize detailed interaction relationships between all genes in the area. For single binary tracks, the node-link diagram option is also available for network areas with less than a pre-specified number of present genes to visualize detailed interaction relationships between present genes in the area. Data in the single continuous and single binary tracks for individual genes are used to determine node colors in the node-link diagrams. In addition, two tracks can also be co-visualized in a node-link diagram in which node and edge colors are determined by data in one of the tracks, respectively.

As described earlier, data in the portal are processed at four different levels. Users can usually start from a higher-level summary track, and when an interesting network area is located, corresponding lower-level data can be retrieved to gain more detailed information. For example, when a network area with enriched number of mutated genes is located, the corresponding mutation matrix can be retrieved to investigate mutation status for these genes in individual samples.

### 2.4 Integrative analysis features

The portal enables data integration at three levels and provides various features for each level of data integration.

First, comparable data generated at the same omics level from multiple independent studies can be integrated. For example, multiple groups have performed gene expression profiling studies to identify genes significantly correlated with patient survival time (Jorissen *et al*. 2009; Reid *et al*., 2009; Smith *et al*., 2010; Staub *et al*., 2009). We used the order statistics method (Wang *et al*., 2013) to integrate data from these studies, and the pre-computed results are readily available as a track. Similarly, integrated tracks for differential expression between stages I and IV tumors or between MSI and MSS tumors in multiple gene expression studies have also been pre-computed, respectively. In addition to retrieving pre-computed results, users can also use the filtering feature to select genes from individual studies and then use the Boolean operation feature (i.e. interactive Venn diagram) to perform simple data integration on the fly.

Second, data generated at multiple omics levels can also be integrated. The omics snapshot based on the multi-omics data from the TCGA tumor cohort provides an easy way to achieve such integration by visual examination. Moreover, based on data from the TCGA tumor cohort, correlation between SCNA level and mRNA/protein abundance has been pre-computed for individual genes, which provides a means for prioritizing genes in the SCNA regions. Users can also combine the filtering and Boolean operation features to perform multi-omics data integration on the fly. For example, genes that are epigenetically silenced and also down regulated at protein level can be identified through such integration. Similarly, genes that are mutated or deleted in CRC can also be identified.

Moreover, experimental data can be integrated with network or pathway information. Experimental data can be either an individual omics dataset or an integrated dataset resulted from abovementioned methods. For network analysis, NetGestalt has implemented a module-based approach and a direct neighborhood approach. In the module-based approach, enrichment analysis is performed for a single binary or a single continuous track against protein–protein interaction network modules at all hierarchical levels based on the Fisher’s exact test or the Kolmogorov-Smirnov test, respectively. In addition to the network modules, enrichment analysis can also be performed against the GO or pathway gene sets. In the direct neighborhood approach, genes in a single binary track are considered ‘seeds’. For individual genes in the network, all direct neighbors are identified and evaluated for the enrichment of the seeds using the Fisher’s exact test. To prioritize the seed genes, the analysis can be focused on only the seed genes. To expand seed genes to include other potentially interesting genes, the analysis can be focused on only the non-seed genes. When a single binary track contains 10 or fewer genes, NetGestalt allows easy retrieval of all direct neighbors of the genes.

### 2.5 Software implementation

NetGestalt is developed using the Ajax (Asynchronous JavaScript and XML) technology with JavaScript on the client side and PHP/Python/C++/R on the server side. A complete list of features implemented in NetGestalt is provided in Supplementary Table S2. All data described in this article can be accessed at http://www.netgestalt.org. A detailed usage guide is available in the Supplementary Manual.

## 3 Case studies

In this section, we use three case studies to illustrate some of the possible applications of the NetGestalt CRC portal.

### 3.1 Retrieving information for a gene

One simple usage of the portal is to retrieve information for a gene of interest, and we will use *KRAS*, one of the best-studied proto-oncogene in CRC, as an example. After retrieving the omics snapshot for visualization (Fig. 2a) and zooming into the *KRAS* gene (Fig. 2b), we can immediately see that *KRAS* is significantly mutated in CRC, does not have significant copy number or methylation changes, and is down-regulated at both mRNA and protein levels in tumors compared with normal colon biopsies. As *KRAS* is an oncogene, this is somewhat counterintuitive. To better understand the KRAS protein expression pattern, corresponding proteomics data can be retrieved and visualized. Although the KRAS protein abundance is indeed lower in tumors compared with normal controls, it is obvious that tumors with mutant *KRAS* have higher KRAS abundance compared with those without mutant *KRAS* (Fig. 2c), suggesting the dependence of CRC tumors on the mutant but not wild-type KRAS protein. In addition to acquiring information for *KRAS*, NetGestalt also allows a quick overview of information for genes in this network neighborhood. Several other significantly mutated genes, including *BRAF*, *NRAS* and *PIK3CA*, reside in this neighborhood (Fig. 2b), which suggest the functional importance of this network neighborhood and also reinforces the critical role of *KRAS* in colon cancer. Sample-level mutation information for these genes can be further retrieved and visualized (Fig. 2d). Although many genes are frequently mutated in the hypermutated tumors, only the abovementioned genes are frequently mutated in the non-hypermutated tumors. Moreover, a node-link diagram depicting detailed interaction relationships between genes in the neighborhood can also be created (Fig. 2e). Although many databases provide gene-centric data retrieval, the portal enables unparalleled easy, intuitive, and flexible access to multi-omics data within a biological network context.

### 3.2 Retrieving information for a gene set

**Fig. 3.**
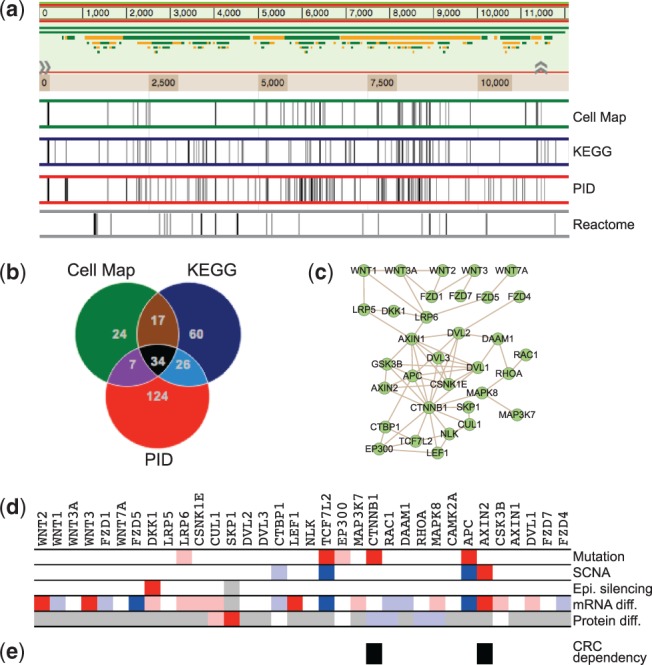
Retrieving information for the WNT signaling pathway. **(a)** Genes in four WNT signaling pathways annotated by the Cancer Cell Map, KEGG, PID and Reactome databases, respectively are visualized as four single binary tracks. **(b)** A Venn diagram comparison of the WNT signaling pathways annotated by the Cancer Cell Map, KEGG and PID databases identified 34 common genes. **(c)** Among these common genes, 33 genes form a connected protein-protein interaction network that covers the well-known components of the WNT signaling pathway. The omics snapshot **(d)** and the CRC cell line specific essential gene track **(e)** depict CRC related molecular alterations and CRC dependency of these 34 genes

Another common usage of the portal is to retrieve information for a set of genes defined in a single binary track, and we will use the WNT signaling pathway as an example. A search for ‘WNT’ in the portal returned four WNT pathways in the Cancer Cell Map, KEGG, PID and Reactome databases, respectively. Co-visualization of these pathway annotations showed that the Reactome annotation is clearly different from all the others (Fig. 3a). A Venn diagram comparison of the remaining three annotations showed a moderate overlap, with only 34 common genes out of the total of 292 genes annotated by these three databases (Fig. 3b). This type of information, although very important to the users, cannot be easily retrieved from other pathway databases or analysis tools. Among the 34 common genes, 33 form a connected protein–protein interaction network that covers the well-known components of the WNT signaling pathway (Fig. 3c). After adding other tracks, data for these genes can be easily retrieved and visualized using the filtering feature. As shown in Figure 3d, both *TCF7L2* and *APC* are mutated, deleted, and under-expressed in CRC tissues. *AXIN2* is amplified and over-expressed in CRC tissues; it also shows a CRC lineage-specific dependency based on cell line data from the Achilles project (Fig. 3e). *CTNNB1* is mutated in CRC tissues and shows CRC lineage-specific dependency (Fig. 3d and e). Molecular alterations for other genes can also be easily spotted. Thus, NetGestalt provides a convenient way to annotate gene sets based on multi-omics data.

### 3.3 Prioritizing epigenetically silenced genes

**Fig. 4.**
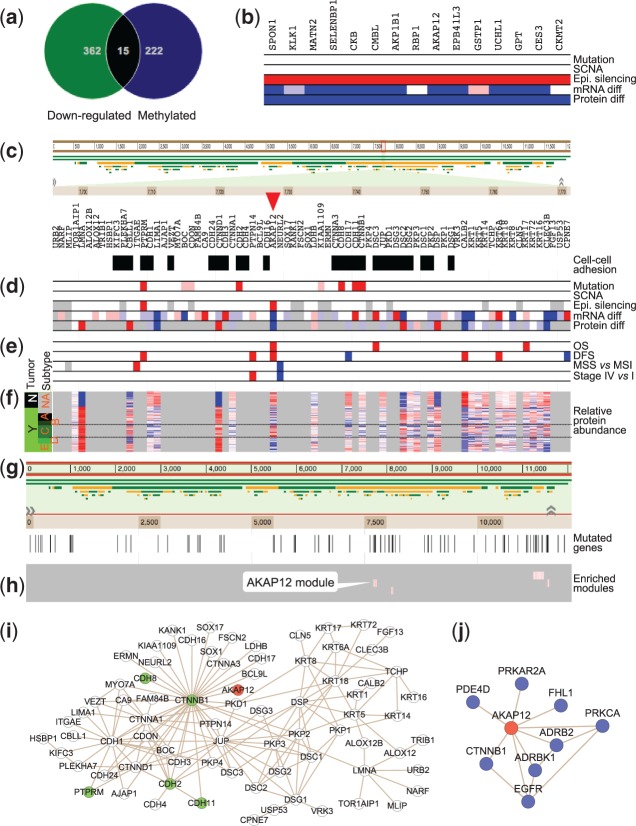
Prioritizing and interpreting epigenetically silenced genes. **(a)** Venn diagram analysis identified 15 epigenetically silenced genes that were also significantly down-regulated at the protein level in CRC. **(b)** Using the filtering feature, an omics snapshot for the 15 genes was created. **(c)** AKAP12 resides in a network module with 76 genes, and the module is significantly enriched with cell-cell adhesion genes. **(d)** The omics snapshot revealed five significantly mutated genes in this module. **(e)** The clinical relevance snapshot showed that higher mRNA level of *AKAP12* was significantly associated with decreased OS and DFS time. **(f)** A detailed view of the proteomics data showed that compared with normal colon tissue, AKAP12 is down-expressed in most tumor samples except for subtype C tumors. **(g)** Non-random distribution of the 92 significantly mutated genes in the network. **(h)** These genes are significantly enriched in four network modules, including the AKAP12 containing module. The four modules are colored in pink and mutated genes in these modules are colored in red. **(i)** The involvement of AKAP12 in this module is primarily mediated through the interaction with CTNNB1. **(j)** All direct interaction partners of AKAP12

The TCGA CRC study identified hundreds of epigenetically silenced genes. Here we use the portal to prioritize and interpret these epigenetically silenced genes. After retrieving both epigenetically silenced genes and genes significantly down-regulated at the protein level in CRC, a Venn diagram analysis identified 15 epigenetically silenced genes that were repressed at the protein level (Fig. 4a). Using the filtering feature, an omics snapshot for the 15 genes was created (Fig. 4b). As expected, most of these genes were also significantly down-regulated at the mRNA level. Using *AKAP12* as an example, we illustrate how to use the portal to find evidence to support the functional importance of the gene. AKAP12 resides in a network module with 76 genes, and the GO biological process enrichment analysis showed that the module is significantly enriched with cell–cell adhesion genes (26 out of the 76 genes, FDR = 4.48e-24, enrichment ratio = 18.3) (Fig. 4c).

Notably, the omics snapshot revealed five significantly mutated genes in the module (Fig. 4d). To test whether this module is significantly enriched with mutated genes, we performed network module enrichment analysis for the 92 significantly mutated genes (Fig. 4g), and the AKAP12 containing module was one of four enriched modules (Fig. 4h, FDR = 0.006, enrichment ratio = 8.6). As shown in Figure 4i, the involvement of AKAP12 in this module is mediated through the interaction with CTNNB1. The portal also allows quick identification and visualization of all direct interaction partners of AKAP12, which includes other well-known cancer genes such as EGFR and PRKCA (Fig. 4j). Network-based analysis thus suggests potential importance of AKAP12 in cancer. The clinical snapshot showed that higher mRNA level of *AKAP12* was significantly associated with decreased OS and DFS time (Fig. 4e). Because *AKAP12* is hypermethylated and under-expressed in CRC compared with normal colon tissues, this was unexpected. A detailed view of the proteomics data showed that compared with normal colon tissue, AKAP12 is down-expressed in most tumor samples except for subtype C tumors (Fig. 4f), which have been associated with epithelial-mesenchymal transition and possibly poor outcome (Zhang *et al*., 2014). A literature review indicates that hypermethylation of *AKAP12* and accompanied under-expression of the gene has been noted in CRC (Liu *et al*., 2010; Mori *et al*., 2006) as well as other human cancers (Choi *et al*., 2004; Flotho *et al*., 2007; Heller *et al*., 2008; Jin *et al*., 2008; Tessema *et al*., 2008). It has been shown that *AKAP12* mRNA was under-expressed in 31 out of 45 (69%) colorectal carcinoma tissues and methylation of *AKAP12* promoter region was detected in 35 (78%) of these tissues (Liu *et al*., 2010). Similarly, we found that the AKAP12 protein was under-expressed in most of the tumors in the TCGA cohort. However, our analysis also reveals that AKAP12 under-expression is likely not involved in the poor-prognosis subtype C tumors, a finding that may have an important clinical implication.

## 4 Discussion

The NetGestalt CRC portal brings together a comprehensive collection of CRC-related omics data. It not only provides easy query and visualization of the data but also enables efficient data integration within a single omics data type, across multiple omics data types, and over biological networks. Our case studies demonstrate the key features that distinguish NetGestalt from other related tools.

Compared with typical network visualization tools, NetGestalt allows multi-scale representation and navigation of the data and enables simultaneous visualization of different types of data to facilitate data integration. Network visualization and analysis tools such as Cytoscape (Shannon *et al*., 2003), VisANT (Hu *et al*., 2009) and Osprey (Breitkreutz *et al*., 2003) have already become indispensible tools in systems biology studies. Nevertheless, node-link diagrams do not scale well with increasing network size and data complexity. First, when the number of nodes goes beyond a few hundreds, neither individual nodes and edges nor the modular structure of the network can be clearly visualized. Second, simultaneous visualization of multiple datasets in a network is inevitably problematic because of a lack of space. Efforts have been made to collapse all members of a module into a singe ‘meta-node’ (Hu *et al*., 2009), extend the two-dimensional representation into three-dimension (Pavlopoulos *et al*., 2008), use animation to visualize data from multiple conditions (Shannon *et al*., 2003), use CircleMaps to represent data associated with individual nodes (Wong *et al*., 2013) or arrange multiple versions of the same network in a grid (Barsky *et al*., 2008). Despite enormous research efforts, it remains challenging to use networks as a framework for the visualization and integrative analysis of large and heterogeneous datasets. NetGestalt offers one solution to provide a network-centric view of multidimensional cancer omics data.

Although the NetGestalt interface shares many similarities with that of the genome browsers, they are conceptually different and complement each other. Genome browsers are effective in integrating genome-anchored information such as gene structure annotation and copy number variation (Kent *et al*., 2002). Nevertheless, they are not designed to reveal functional relationships among gene products (Nielsen *et al*., 2010). NetGestalt brings functionally related genes together to facilitate the interpretation and integration of multi-omics data within the context of biological networks.

Another unique aspect of NetGestalt is the enabling of seamless connections among the data query, visualization, and integration features, which can be used iteratively in different combinations to serve the needs of the users, maximizing the synergy among omics data, human visualization, and quantitative analysis. As a result, the portal can be used for both hypothesis generation and hypothesis testing, or a combination of both. Moreover, users can also upload their own datasets to the system for integrative analysis.

The current version of the portal only includes three human protein–protein interaction networks. Genes not included in the networks are not included for visualization and analysis in the portal. In the future, we will add functional association networks (e.g. STRING, Szklarczyk *et al*., 2011) that do not require physical interaction in order to further improve gene coverage. Moreover, it has been shown that tissue-specific networks are more functionally relevant (Guan *et al*., 2012; Magger *et al*., 2012). We will consider adding context-specific networks to the portal. Meanwhile, users can analyze the datasets using their own networks, which can be easily uploaded to the system.

Using CRC as a paradigm, we have demonstrated the power of NetGestalt in facilitating easy access and interpretation of multidimensional cancer omics data. A natural extension is to expand the portal to cover data for other cancer types. Analysis across tumor types, as demonstrated by the Pan-Cancer initiative (Cancer Genome Atlas Research *et al*., 2013), has started to provide novel biological and clinical insights to human cancer. A NetGestalt pan-cancer portal will complement existing efforts within the Pan-Cancer initiative to facilitate the discovery of commonalities, differences and emergent themes across cancer types at gene, pathway, and network levels.

## Funding

This work was supported by contract 13XS029 from Leidos Biomedical Research, Inc. It was conducted in part using the resources of the Advanced Computing Center for Research and Education at Vanderbilt University, Nashville, TN. We thank Peter Straub and Jason Castellanos for proofreading the manuscript.

*Conflicts of interest*: none declared.

## Supplementary Material

Supplementary Data
