# Supplementary material for: Empowering biologists with multi-omics data: colorectal cancer as a paradigm
Source: Bioinformatics. 2014 Dec 18;31(9):1436–43. doi: 10.1093/bioinformatics/btu834 (PMC4410657; doi:10.1093/bioinformatics/btu834)
Supplement: Supplementary Data [file supp_31_9_1436__index.html]

Empowering biologists with multi-omics data: colorectal cancer as a paradigm — Empowering biologists with multi-omics data: colorectal cancer as a paradigm — Supplementary Data 

# Empowering biologists with multi-omics data: colorectal cancer as a paradigm

## Supplementary Data

files

**Files in this Data Supplement:**

- Supplementary Data - pdf file
