# Supplementary material for: Empowering biologists with multi-omics data: colorectal cancer as a paradigm
Source: Bioinformatics. 2014 Dec 18;31(9):1436–43. doi: 10.1093/bioinformatics/btu834 (PMC4410657; doi:10.1093/bioinformatics/btu834)
Supplement: Supplementary Data [file supp_btu834_Zhu_NGCRC_Supplemental_combined.pdf]

Empowering biologists with multi-omics data: colorectal cancer as a paradigm

Jing Zhu[1†], Zhiao Shi[2,3†], Jing Wang[1], Bing Zhang[1,4*]

[1]Department of Biomedical Informatics, [2]Advanced Computing Center for Research and Education, [3]Department of Electrical Engineering and Computer Science, [4]Department of Cancer Biology, Vanderbilt University, Nashville, Tennessee, USA.

SUPPLEMENTARY METHODS, TABLES AND USER MANUAL

SUPPLEMENTARY METHODS

Data sources

Genomic, epigenomic, transcriptomic, and clinical data for the TCGA CRC cohort were acquired from the TCGA publication (TCGA Research Network, 2012) or downloaded from the TCGA Firehose pipeline on Aug 20, 2013, based on the latest TCGA production runs. The proteomics data for this tumor cohort were described in our recent publication (Zhang, et al., 2014). The portal also included mRNA expression data from five GEO (Gene Expression Omnibus) CRC cohorts with survival information (GSE17536, GSE17537, GSE12945, GSE16125 and GSE14333). In addition to data from tumor specimens, we also collected data from CRC cell lines including drug response data, genomic and transcriptomic data from CCLE (Cancer Cell Line Encyclopedia) project (Barretina, et al., 2012) and shRNA screen data from the Achilles project (Cheung, et al., 2011).

The human protein-protein interaction networks are based on data from the Human Protein Reference Database (Keshava Prasad, et al., 2009) and the iRef database (Turner, et al., 2010) respectively. The former is a single source manually curated database that includes 9198 proteins and 36707 interactions whereas the latter consolidates data from 10 public databases and includes 12112 proteins and 91914 interactions. The pathway data are from Cancer Cell Map (http://www.netpath.org/), HumanCyc (Romero, et al., 2005), KEGG (Kanehisa, et al., 2006), NCI Pathway Interaction Database (Schaefer, et al., 2009), and Reactome (Croft, et al., 2014).

Data processing

When available, gene-level data were downloaded from the above resources and converted to an appropriate NetGestalt data format as described in the Supplementary Manual. When gene-level data is not available, we summarized lower-level data to gene level. For the methylation data generated using Illumina Infinium HumanMethylation27 microarrays, when probes targeting multiple genes were found, the values of this probe

were assigned to each gene. When genes targeted by multiple probes were found, the probe that is most anticorrelated with expression data was selected. If the corresponding expression data are not available, mean values of the multiple probes were used. For gene expression data sets from GEO, in each individual data set, for all probe sets that map to the same gene, the probe set with the largest interquartile range (IQR) was selected to represent the gene. For composite tracks containing multiple samples, sample annotations were processed to the *tsi* format as described in the Supplementary Manual.

Based on the composite tracks and corresponding sample annotations, we performed statistical analyses including the *t*-test, Wilcoxon rank-sum test, Cox regression, and Spearman's test as appropriate. For all the genes, we converted the resulted statistics and *p* values to *sct*s. Based on pre-defined criteria, such as FDR < 0.01 and fold change >2, we filtered these *sct*s to generate *sbt*s. For drug response data from CCLE project, the spearman correlation coefficients between response (activity area) of 24 compounds and gene expression of each gene in available CRC cell lines were computed and converted to *sct*s.

For clinical relevant analysis such as finding markers for overall/disease free survival or signature genes for MSI vs MSS or Stage IV vs Stage I, we used order statistics computed using the method described in (Wang, et al., 2013) to identify makers or signature genes supported by multiple data sets. Taking finding markers for overall survival as an example, signed $-\log_{10}$(order Statistic) values were used to summarize the results from multiple data sets and stored in an *sct*. Large absolute values indicate a high correlation with overall survival as supported by multiple data sets. A positive sign means log2HazardRatio > 0, or correlation with poor overall survival. A negative sign means log2HazardRatio < 0, or correlation with good overall survival. Then, based on pre-defined criteria, such as signed $-\log_{10}$(order Statistic) value ≥ 2 or ≤ -2, we filtered the *sct* to generate two *sbt*s, indicating the markers for poor and good overall survival, respectively.

For each of the three types of data analysis including genomic and proteomic alterations, clinical relevant markers/signature genes, and genes associated with drug sensitivity, the results were summarized as a "snapshot" track. The omics snapshot shows the summary of all CRC related alterations for all the genes based on multi-dimensional omics data including significantly mutated genes, genes in the focal amplification/ deletion regions, epigenetically silenced genes, and genes differentially expressed at mRNA level and protein level. The clinical relevance snapshot shows the summary of clinical relevance for all the genes based on multiple data sets including the markers for poor/good disease free/overall survival, the signature genes for MSI vs MSS and the signature genes for Stage IV vs Stage I. The drug response snapshot shows the spearman correlation between drug response (activity area) of 24 compounds and gene expression in available CRC cell lines.

REFERENCES

Barretina, J., *et al*. The Cancer Cell Line Encyclopedia enables predictive modelling of anticancer drug sensitivity. *Nature* 2012;483(7391):603-607.

Cheung, H.W., *et al*. Systematic investigation of genetic vulnerabilities across cancer cell lines reveals lineage-specific dependencies in ovarian cancer. *Proc Natl Acad Sci U S A* 2011;108(30):12372-12377.

Croft, D., *et al*. The Reactome pathway knowledgebase. *Nucleic acids research* 2014;42(Database issue):D472-477.

Kanehisa, M., *et al*. From genomics to chemical genomics: new developments in KEGG. *Nucleic acids research* 2006;34(Database issue):D354-357.

Keshava Prasad, T.S., *et al*. Human Protein Reference Database--2009 update. *Nucleic Acids Res* 2009;37(Database issue):D767-772.

Romero, P., *et al*. Computational prediction of human metabolic pathways from the complete human genome. *Genome biology* 2005;6(1):R2.

Schaefer, C.F., *et al*. PID: the Pathway Interaction Database. *Nucleic acids research* 2009;37(Database issue):D674-679.

TCGA Research Network. Comprehensive molecular characterization of human colon and rectal cancer. *Nature* 2012;487(7407):330-337.

Turner, B., *et al*. iRefWeb: interactive analysis of consolidated protein interaction data and their supporting evidence. *Database : the journal of biological databases and curation* 2010;2010:baq023.

Wang, J., *et al*. Integrative genomics analysis identifies candidate drivers at 3q26-29 amplicon in squamous cell carcinoma of the lung. *Clin Cancer Res* 2013;19(20):5580-5590.

Zhang, B., *et al*. Proteogenomic characterization of human colon and rectal cancer. *Nature* 2014.

Table S1 Data tracks and sample size in the NetGestalt CRC portal

Track category	Sample source	Sample size	Number of tracks
Omics snapshot	Primary tumor tissue	--	1
Somatic mutation	Primary tumor tissue	224	4
Somatic copy number alteration	Primary tumor tissue	257	4
Epigenetic silencing	Primary tumor tissue	276	2
Differential mRNA expression	Primary tumor tissue	244	6
Differential protein expression	Primary tumor tissue	120	16
Correlation between mRNA and protein	Primary tumor tissue	87	2
Correlation between SCNA and mRNA	Primary tumor tissue	255	2
Correlation between SCNA and protein	Primary tumor tissue	88	2
Clinical relevance snapshot	Primary tumor tissue	--	1
Marker for overall survival	Primary tumor tissue	326	11
Marker for disease free survival	Primary tumor tissue	318	11
Signature gene for Stage IV vs Stage I	Primary tumor tissue	182	15
Signature protein for Stage IV vs Stage I	Primary tumor tissue	25	3
Signature gene for MSI vs MSS	Primary tumor tissue	495	15
Signature protein for MSI vs MSS	Primary tumor tissue	90	3
Drug response snapshot	CRC cell lines	--	1
Predictor of drug sensitivity	CRC cell lines	23	24
Essential genes	CRC cell lines	102	1
Gene Expression	CRC cell lines	61	1
Somatic copy number alteration	CRC cell lines	58	1
Cancer Cell Map	Functional tracks	--	10
GO Biological Process	Functional tracks	--	808
GO Cellular Component	Functional tracks	--	231
GO Molecular Function	Functional tracks	--	383
HumanCyc	Functional tracks	--	267
KEGG	Functional tracks	--	200
NCI	Functional tracks	--	223
Reactome	Functional tracks	--	1108

Table S2 Features implemented in the NetGestalt CRC portal

Feature	Track type	Description
Single track features		
Network analysis (n)*	SBT, SCT	
Module enrichment	SBT, SCT	Identify enriched network modules for a track of interest
Network expansion	SBT	Expand genes in a track to include network neighbors
Gene Prioritization	SBT	Prioritize genes in a track
Gene set enrichment (r)	SBT, SCT	Perform pathway enrichment analysis
Node-link graph, visible range (G)	SBT, SCT, CBT, CCT	Visualize genes in a node-link diagram for all genes in the visible range
Node-link graph, present nodes (g)	SBT	Visualize genes in a node-link diagram for all present genes in the visible range
Data transformation (t)	SCT, CCT	Gene-wise data standardization (sct only); data truncation
Presence-based filtering (F)	SBT	Filter based on genes in a track
Value-based filtering (f)	SCT	Derive an SBT from an SCT by applying numerical filtering
Subtrack annotation (a)	CBT, CCT	Visualize sample meta-data as a companion heat map
Track information (i)	SBT, SCT, CBT, CCT	Retrieve detailed information for a track from the database and display
Export (e)	SBT, SCT, CBT, CCT	Export data
Delete (x)	SBT, SCT, CBT, CCT	Delete track
Multi-track features		
Track comparison	SBTs	Compare multiple tracks in a Venn diagram and create new tracks
Track co-visualization	SBTs, SCTs	Co-visualize two tracks in a node-link diagram
General features		
Browse all tracks	All	Browse all tracks in the database
Search for a track	All	Search for tracks in the database (with search term suggestion)
Upload a track	All	Upload local data in standardized formats for visualization
Upload gene symbols	SBT	Paste gene symbols to generate an SBT
Move a track	All	Rearrange the track order to facilitate visual comparison
Change network	All	Switch between different networks and hierarchical architectures
Upload network	All	Upload user-specific networks in the standardized format (nsm)
Zoom	All	Zoom to a specific module, a user specified region, or around a gene
Pan	All	Pan to left and right

*: Abbreviations used in NetGestalt;
CBT: Composite Binary Track; CCT: Composite Continuous Track; SBT: Single Binary Track; SCT: Single Continuous Track

NetGestalt CRC portal User Manual

May 20, 2014

NetGestalt CRC portal is a web-based resource that allows simultaneous presentation of large scale experimental and annotation data from many sources in the context of biological networks to facilitate data interpretation and hypothesis generation.

The current version of the NetGestalt CRC portal contains: (1) genomic, epigenomic, transcriptomic, proteomic and clinical data based on colorectal cancer (CRC) tumor tissue; (2) drug response data, genomic, transcriptomic data and shRNA screen data based on CRC cell lines; and (3) existing knowledge on protein-protein interaction, pathways databases and Gene Ontology.

Data query and integrative analysis with NetGestalt CRC portal follows a very intuitive workflow:

1. Set a view

To perform analysis in NetGestalt CRC portal, a user should first select a view. Hovering the mouse over the "View" menu (see red box in Figure 1), different choices for setting the view will be shown in a drop-down menu.

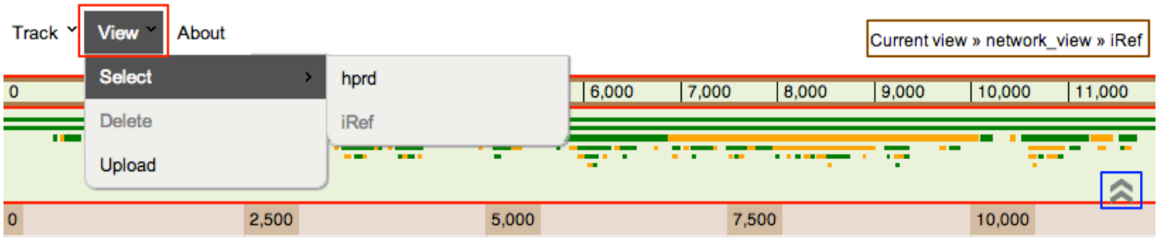

Figure 1. Set a view in NetGestalt CRC portal

a. Select a view

Hovering the mouse over "Select", all views provided by the system will be shown in a menu. The currently active view is shown in grey, while others are shown in black.

The user can select a view by clicking the name. After setting the view, the user can find the name of the selected view and corresponding category at the top-right of the window (see brown box in Figure 1). In the current version, NetGestalt CRC portal contains one category (network_view) and two views under the category: hprd corresponding to the HPRD human protein-protein interaction (PPI) network (http://www.hprd.org/) and iRef corresponding to the iRef human PPI network (http://wodaklab.org/iRefWeb/). In the future, other networks will be added to the network view, and other categories (*e.g.* pathway view and chromosome view) will also be added.

After selecting a "network_view", a one-dimensional layout of network nodes will be shown right below the menu (Figure 1). Bars in different length and thickness represent modules at different hierarchical levels of the network. The thick bars correspond to modules in the best partition of the global network. Modules at this level can be split into smaller ones represented by thin bars. Alternating bar colors (green and orange) are used to help users distinguish neighboring modules. The module information can be hided by clicking the grey "double arrow" button at the right bottom of this region (see blue box in Figure 1).

b. Upload network

Users can also upload their own networks into NetGestalt CRC portal by clicking the "Upload" button below the "Select" button in the menu (see red box in Figure 2). After clicking, an "Upload Network" dialog window will pop up. In this window, a user can click the "Choose File" button to select a local file and then click the "Submit" button to upload the network to NetGestalt CRC portal. **NetGestalt CRC portal only accepts ".nsm" files as network input, which can be generated by the R package NetSAM (http://www.bioconductor.org/packages/release/bioc/html/NetSAM.html).** The NetSAM package as well as the NetSAM manual can be downloaded in the "Upload Network" dialog window (see brown box in Figure 2). After uploading the network, the current view will be automatically switched to the uploaded network view, which can also be found in the drop down menu under "View" and "Select".

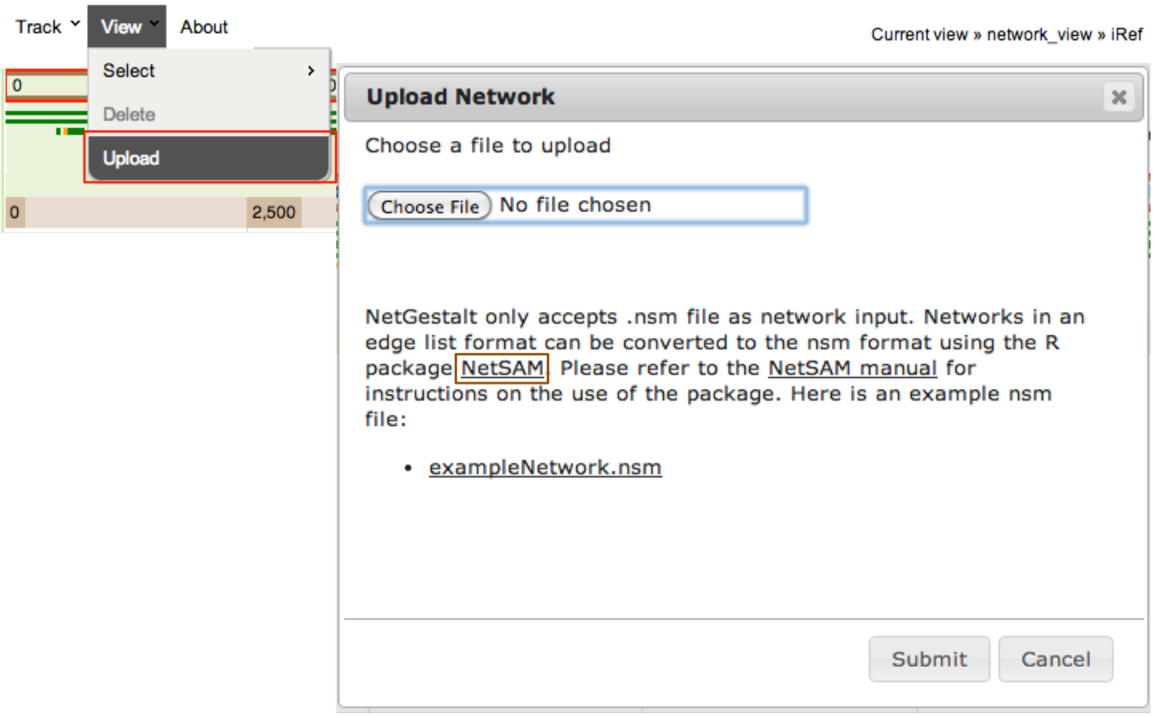

Figure 2. Upload network in NetGestalt CRC portal

c. Delete a view

To delete an uploaded network view, the user has to switch to another network view first

and then click the "delete" button below the "Select" button in the menu under "View".

2. Add tracks

NetGestalt CRC portal provides multiple options for adding tracks to the track viewing area. Hovering the mouse over the "Track" menu (see red box in Figure 3), different options for retrieving and adding tracks of interest will be shown in a drop-down menu.

a. Browse system tracks

A user can click the "Browse System Tracks" button to browse all the tracks included in the NetGestalt CRC portal. In the "Browse All Tracks" dialog, all the tracks are organized into a tree structure.

The user can click the "Expand all" button (see blue box in Figure 3) to expand the tree and select a category of tracks by clicking a leaf node of the tree (such as the leaf node "02_Somatic_Mutation"). Then, a list of tracks contained in the category will be shown in the top-right part of the dialog (see purple box in Figure 3). When clicking one track in the list, such as the "TCGA_COADREAD_Mutation" track (see black box in Figure 3), detailed information associated with the track will be shown in the bottom-right table. Finally, clicking the "+" button (see brown box in Figure 3) will add the track to the track viewing area.

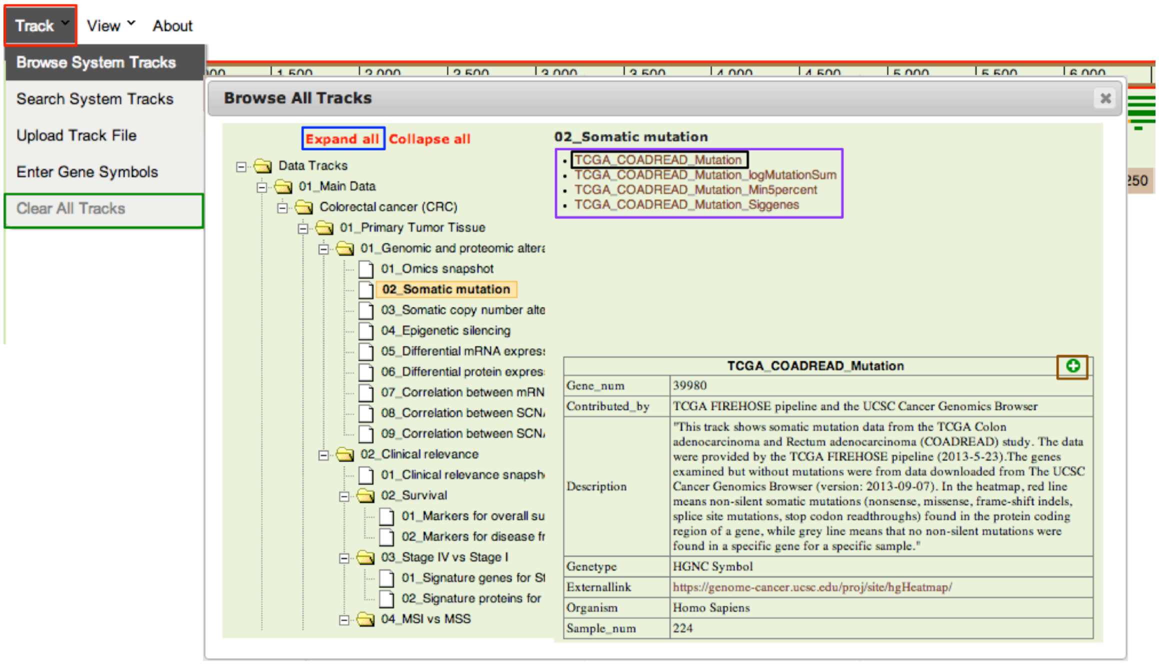

Figure 3. Browse tracks in NetGestalt CRC portal

b. Search tracks

A user can click the "Search System Tracks" button below the "Browse System Tracks" button to search for tracks of interest in the database. After clicking the "Search System Tracks" button, a "Search system tracks" dialog will pop up. The user can input a key

word in the box (see red box in Figure 4). NetGestalt CRC portal will list the names of all matching tracks below the box. Hovering the mouse over the "i" button of a track will display the detailed information about the track. Finally, the track can be added to the track viewing area by clicking the "+" button.

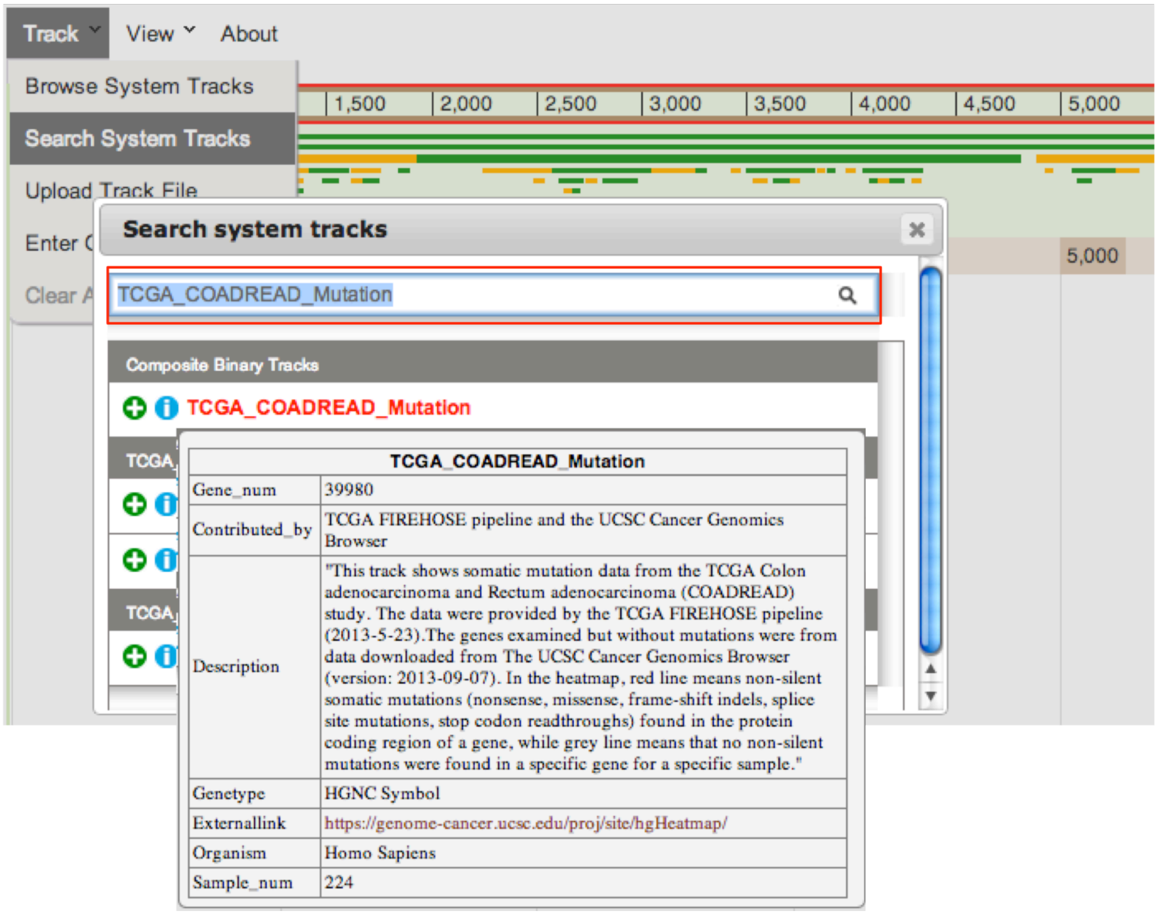

Figure 4. Search tracks in NetGestalt CRC portal

c. Upload tracks

Users can upload their own data into NetGestalt CRC portal by clicking the "Upload Track File" button below the "Search System Tracks" button (see red box in Figure 5). In the "Upload Track" dialog, the user can click the "Choose File" button to select a local file and then click the "Submit" button to upload the file to NetGestalt CRC portal. **For network analyses based on the human PPI networks in the system and the GO/pathway-based gene set enrichment analysis, the current version of NetGestalt CRC portal only works for human genes and only supports gene symbol as gene identifier. If the data is not based on gene symbol, please map other gene identifiers to gene symbols before uploading the data into NetGestalt CRC portal.** For network analyses based on user-uploaded networks, the user can use any type of IDs matched with the IDs in the user-uploaded data tracks. NetGestalt CRC portal supports five types of track files corresponding to different data types. Please see below for file preparation guidelines. Examples for the five types of track files can be downloaded from the

"Upload Track" dialog (see blue box in Figure 5).

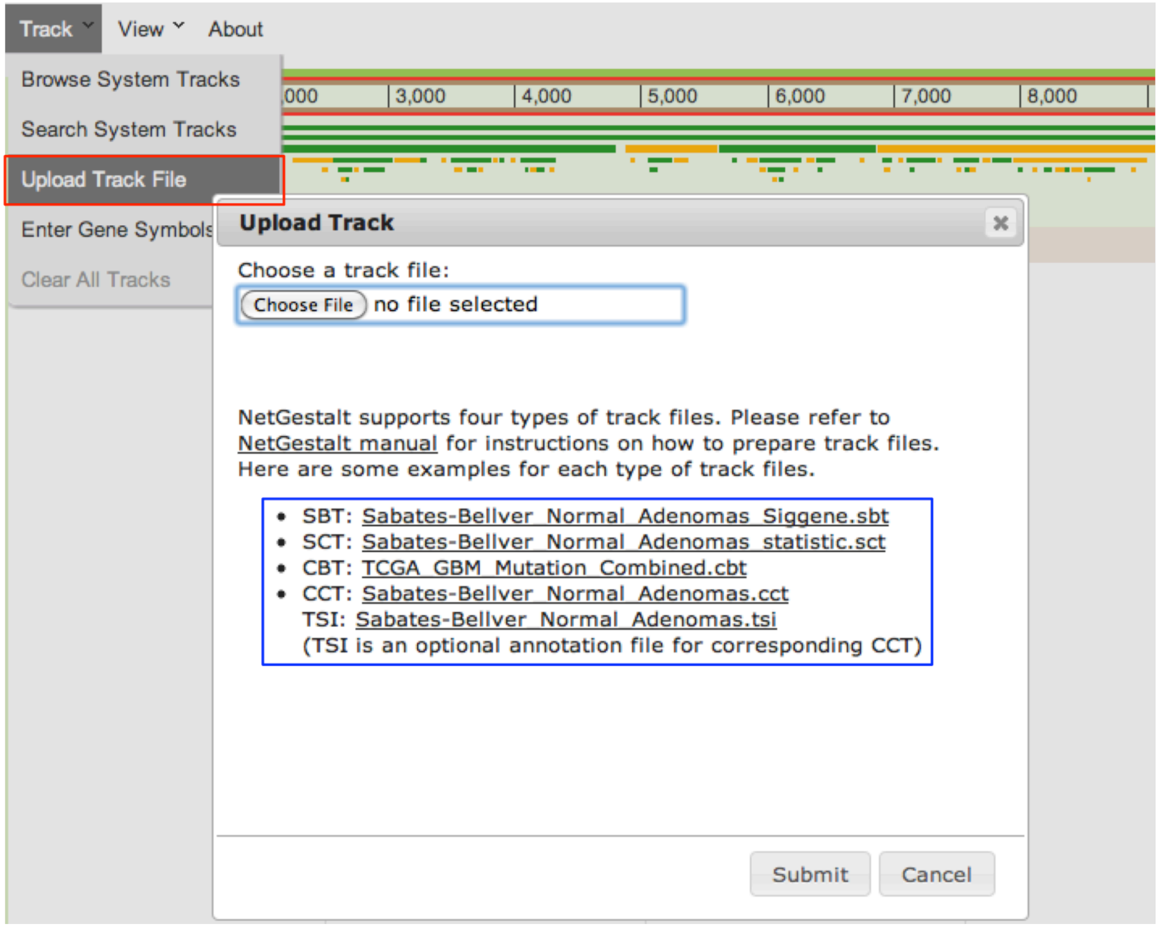

Figure 5. Upload tracks in NetGestalt CRC portal

(i) **Composite continuous track (CCT) file**

Definition: a CCT file (.cct) is a tab-delimited text file that contains data for a composite track with multiple related sub-tracks with continuous data (e.g. microarray gene expression data for samples from the same data set). The file name will be used as the track name.

File format description: a CCT file is a data matrix in which each row represents a gene and each column represents a sample. The first column lists the gene ids (e.g. gene symbols) and the first row lists the sample names. Two or more data columns (sub-tracks) are required. Columns must be separated by tab. Each cell in the matrix is a continuous value for corresponding gene and sample. Missing values are represented by NA. Data for different samples must be comparable (i.e. properly normalized). Duplicated row names or column names are not allowed. No special characters for row or column names. Cells in red should not be changed.

Example:

GeneSymbol	Sample1	Sample2	Sample3	Sample4
Gene1	0.025	-0.55	-1	0.095
Gene2	-0.077	0.069	0.64	0.18
Gene3	-0.47	1	0.87	-0.88
Gene4	-0.71	-0.19	0.33	-0.45

(ii) Composite binary track (CBT) file

Definition: a CBT file (.cbt) is a tab-delimited text file that contains data for a composite track with multiple related sub-tracks with binary data (e.g. mutation status for genes in multiple samples). The file name will be used as the track name.

File format description: same as the CCT file except that each cell in the matrix is a binary value for corresponding gene and sample.

(iii) Track sample information (TSI) file

Definition: a TSI file (.tsi) is a tab-delimited text file that contains the sample information for a CCT or CBT track. This file is an optional sample annotation file for the matching CCT/CBT track file, and it should be uploaded together with the CCT/CBT file.

File format description: a TSI file (.tsi) is a data matrix in which each row represents a sample and each column represents one feature of the samples. Sample features can be divided into three data types including binary data (BIN, *e.g.*, mutation status), categorical data (CAT, *e.g.*, tumor stage) and continuous data (CON, *e.g.*, age). Binary data do not have to be 0/1 but must contain exactly two categories (e.g. yes/no or tumor/normal). The first column lists the sample names. Sample names must match exactly those in the corresponding CCT or CBT track. The first row lists the feature names; and the second row indicates the data type for each feature (must be one of the following: BIN, CAT or CON). Each cell in the matrix is a value for corresponding sample and feature. Missing values are represented by NA. Duplicated row names or column names are not allowed. No special characters for row or column names. Cells in red should not be changed.

Example:

Barcode	Age	Anatomic neoplasm subdivision	Colonpolyps present
data_type	CON	CAT	BIN
TCGA-A6-2670-01	45	sigmoid colon	0
TCGA-A6-2671-01	85	sigmoid colon	0
TCGA-A6-2672-01	82	transverse colon	0
TCGA-A6-2674-01	71	sigmoid colon	0
TCGA-A6-2675-01	78	sigmoid colon	0
TCGA-A6-2676-01	75	cecum	0
TCGA-A6-2677-01	68	cecum	0
TCGA-A6-2678-01	43	transverse colon	0
TCGA-A6-2679-01	73	ascending colon	0
TCGA-A6-2680-01	72	hepatic flexure	0
TCGA-A6-2681-01	73	cecum	0

(iv) Single continuous track (SCT) file

Definition: a SCT file (.sct) is a tab-delimited text file that contains statistical analysis results derived from a data set (e.g. mean, median, sum, variance, t-statistic, p value).

File format description: an SCT file contains at least two columns: the first column with gene ids (e.g. gene symbols) and the second column with statistic scores for the genes. Up to three statistic scores (three columns) can be included in an SCT file. Columns must be separated by tab. The first row of the file lists the track names. We suggest that a track name should contain some descriptions about the corresponding statistic score. For fold changes, it is recommended to perform a log2 transformation. For p values, it is recommended to perform a –log10 transformation and add the direction of change to the transformed p values (e.g. signed p values). Missing values are represented by NA. Duplicated row names or column names are not allowed. No special characters for row or column names. Cells in red should not be changed.

Example:

GeneSymbol	TrackName1	TrackName2
Gene1	-2.2	-4.8
Gene2	-1.3	-1.5
Gene3	5.3	7.6
Gene4	1.1	0.7

(v) Single binary track (SBT) file

Definition: a SBT file (.sbt) is a tab-delimited text file that contains lists of genes in separate rows (e.g. significant genes from differential expression analysis).

File format description: an SBT file contains track name, track description and gene ids (e.g. gene symbols) in the track. Each row represents a track and columns are separated by tab. Up to three tracks (three rows) can be included in an SBT file. To enable meaningful enrichment analysis, the user can include in the first row an “All” track that contains the reference gene symbols for the tracks in the SBT file (e.g. all genes on the microarray platform from which the differentially expressed genes were identified). If this information is not provided, enrichment analysis will be based on all genes in the network. If the “All” track is provided, all genes in the other tracks should be included in the “All” track. No special characters for Track names. Cells in red should not be changed.

Example:

All	Description	GeneSymbol_11	GeneSymbol_21	GeneSymbol_22
TrackName1	Description1	GeneSymbol_11		
TrackName2	Description2	GeneSymbol_21	GeneSymbol_22	

NetGestalt CRC portal uses the following rules to determine the file type of a given file:
(1) Use the file extension to determine the file type. Ignore the .txt file extension. (For example, both test.sbt and test.sbt.txt are treated as a SBT file.)
(2) If that fails, NetGestalt CRC portal cannot determine the file type and displays an error message.

The maximum upload file size is 20MB.

d. Enter gene ids as a track

To enter gene ids (e.g. gene symbols) and add them as a new SBT in NetGestalt CRC portal, the users can click the “Enter gene symbols” button in the “Track” menu (see red box in Figure 6) and then enter or paste gene symbols in the “Enter gene symbols” dialog. The input will be automatically separated into valid gene symbols (i.e. gene symbols included in the current view) and invalid ones. The total number of valid gene symbols will be shown at the bottom of the window. Clicking the “GO” button, entering a track title in the opened dialog, and then clicking the “Add” button will add the valid gene symbols as a new SBT (Figure 6).

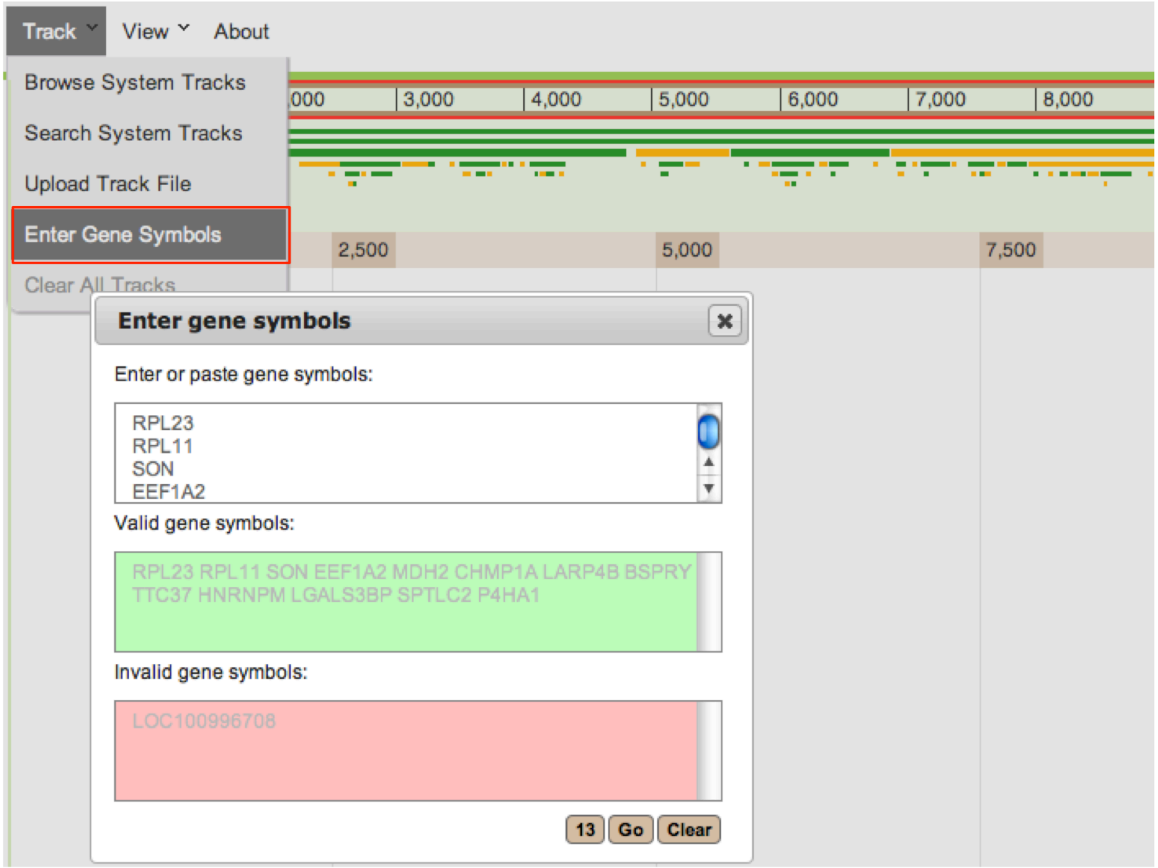

Figure 6 Add gene symbols as a track in NetGestalt CRC portal

3. Visualize Tracks

NetGestalt CRC portal uses different methods to visualize different types of tracks.

a. CCT track

NetGestalt CRC portal visualizes a CCT track with a heat map with colors ranging from blue to red. The first track in Figure 7 is a CCT track representing a gene expression data containing 222 CRC samples and 22 normal samples. When hovering the mouse over the heat map plot, NetGestalt CRC portal will show the gene id, sample index and sample name at the corresponding position.

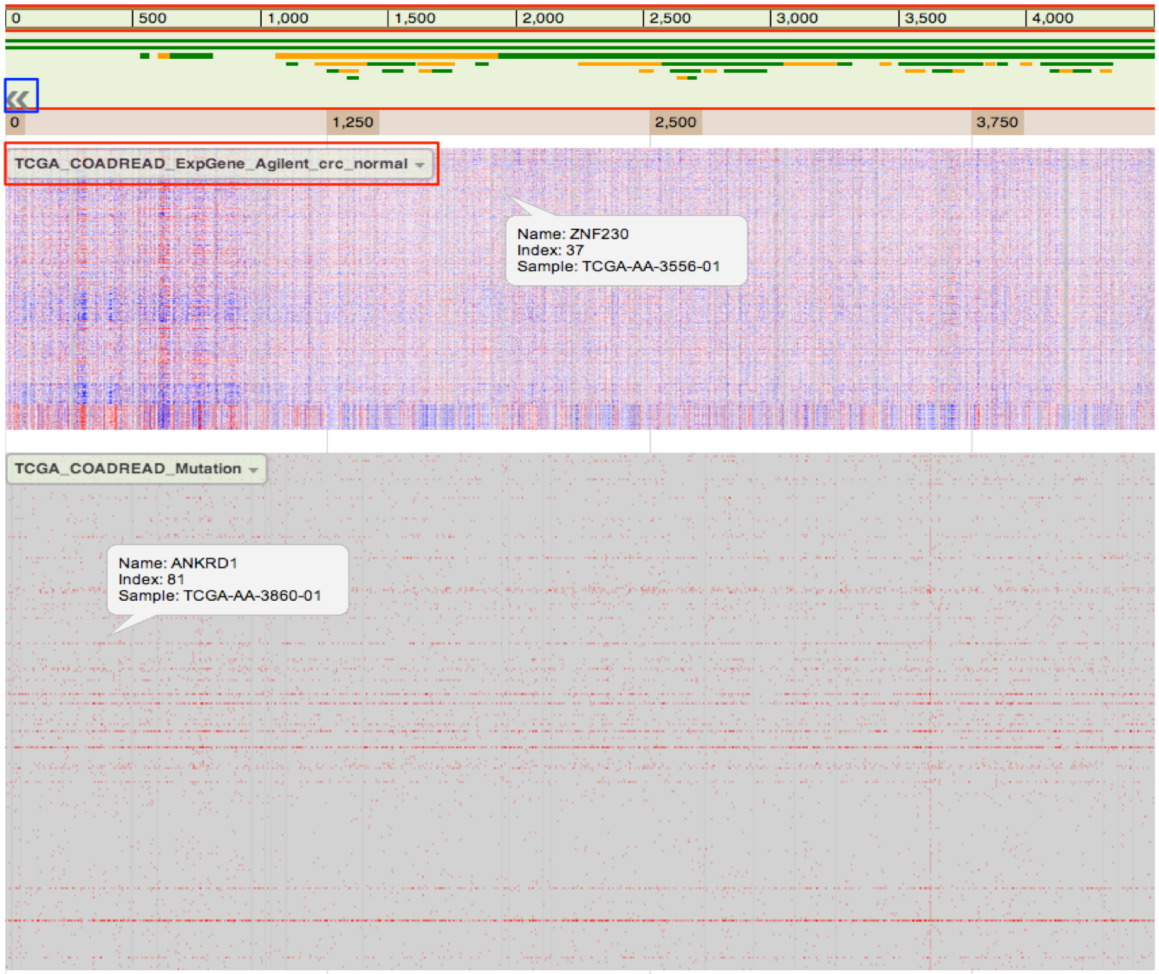

Figure 7. Visualize CCT and CBT tracks in NetGestalt CRC portal

b. CBT track

NetGestalt CRC portal visualizes a CBT track with a heat map of two colors (red and grey). The second track in Figure 7 shows a CBT track representing a TCGA COADREAD somatic mutation data containing 224 CRC samples. Hovering the mouse over the heat map plot will show the gene symbol, sample index and sample name at the corresponding position.

c. SCT track

NetGestalt CRC portal visualizes a SCT track with a bar plot. The first track in Figure 8 is an SCT track containing log fold changes of gene expression between CRC samples and normal samples based on TCGA dataset. Hovering the mouse over the bar plot, NetGestalt CRC portal will display the gene symbol and statistic value at the corresponding position.

d. SBT track

NetGestalt CRC portal visualizes a SBT track with a barcode plot. The second track in Figure 8 is an SBT track representing significantly mutated genes based on TCGA

somatic mutation dataset. Hovering the mouse over the plot will display gene symbol at the corresponding position.

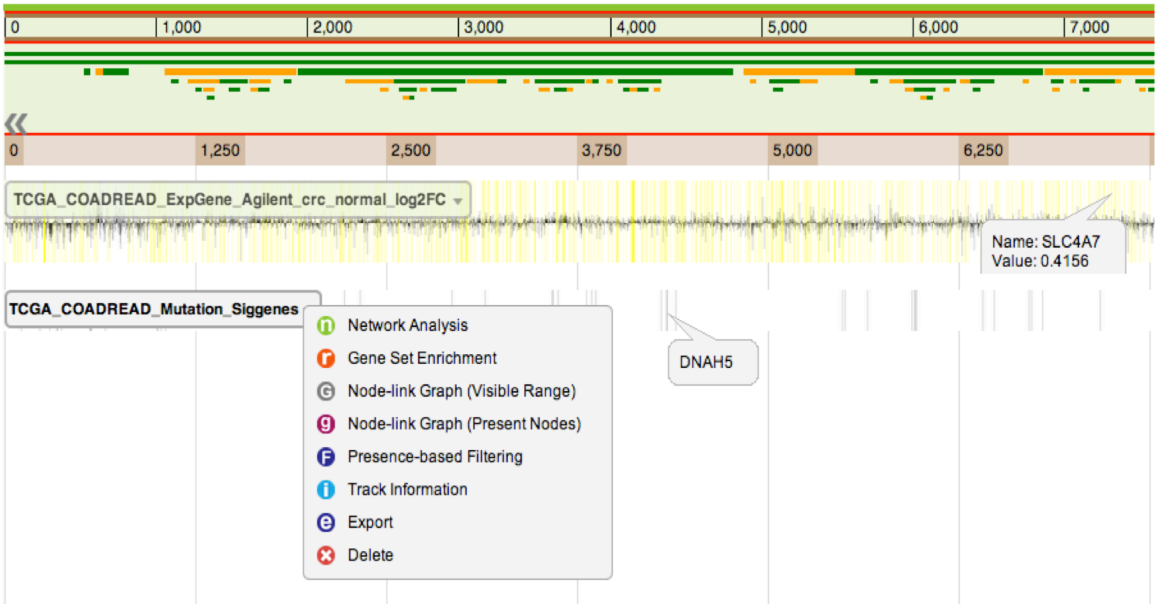

Figure 8. Visualize SCT and SBT tracks in NetGestalt CRC portal

After adding the tracks into the track viewing area, NetGestalt CRC portal automatically shows the track name on the top-left of the track (see red box in Figure 7). User can click the "double arrow" button located above the top-left of the first track to hide these track names (see blue box in Figure 7). Dragging a track name can change the vertical position of the track (not supported by IE).

Hovering the mouse over a track name, all track manipulation and analysis features will be shown in a drop-down menu (Figure 8).

When clicking the "i" button, a table containing detailed information about the track will be shown. Clicking the "e" button will export the track data. Clicking the "x" button will remove the corresponding track from the viewing area. We will introduce other buttons in the section "Analyze tracks". The user can also click the "Clear all tracks" button in the "Track" menu to remove all the tracks from the track viewing area (see green box in Figure 3).

4. Zoom in (out) tracks

NetGestalt CRC portal provides three methods to visualize tracks at different scales.

a. Click bars representing predefined modules

NetGestalt CRC portal uses horizontal bars to represent network modules (sub-networks) at different hierarchical levels. The lengths of the bars correspond to the size of the modules. For a specific hierarchical level, genes within a module are highly connected

whereas genes from different modules are loosely connected. Most of the predefined network modules have been demonstrated to be functionally, spatially or dynamically homogeneous (Shi, et al., 2013). These modules can help users to easily associate subnetworks with experimental data. User can click the corresponding bar to zoom into a module for further analysis (see Figure 9). A track of gene symbols will appear at the top of the track viewing area when it is completely zoomed in (see red box in Figure 9). To zoom out, the user can click the bar at root level.

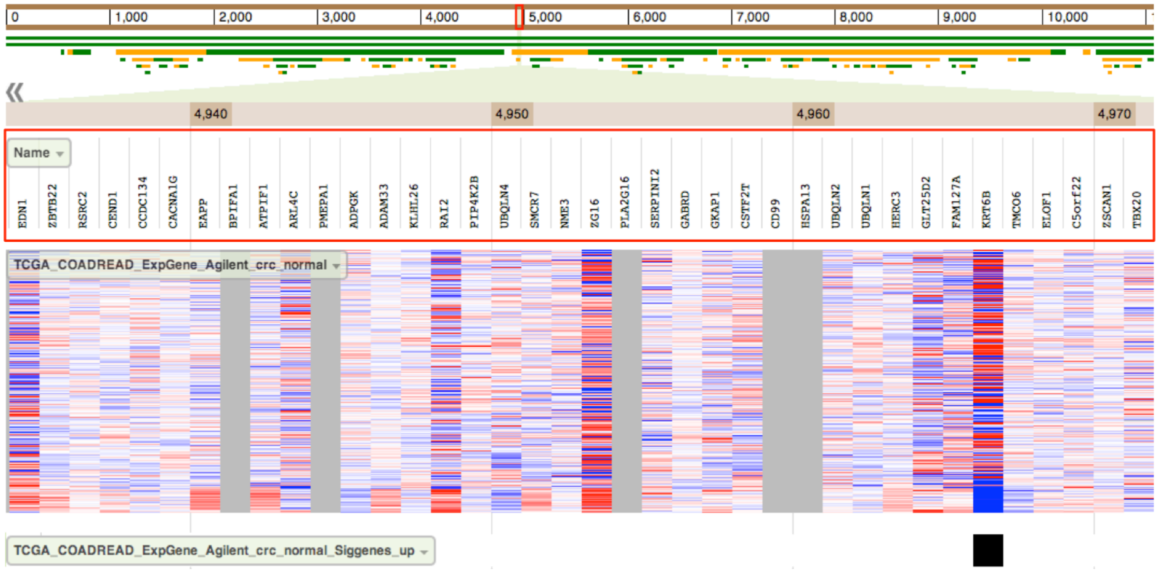

Figure 9. Click bars representing predefined modules to zoom in the tracks

If a user is interested in a region that is not represented by a predefined module, NetGestalt CRC portal provides two additional zoom-in methods for visualizing any regions of the one-dimensionally ordered network (See below).

b. Alt+drag

A user can drag the mouse across a region of interest while holding the 'Alt' key down (see Figure 10).

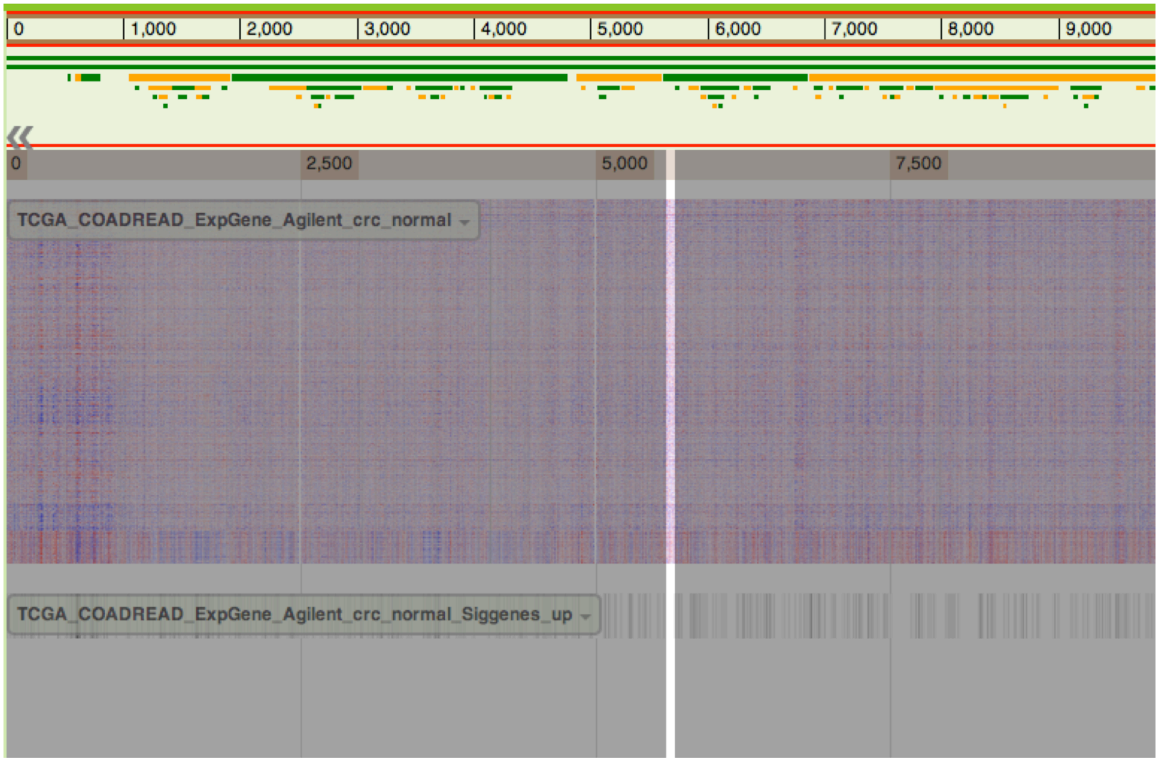

Figure 10. Zoom in the tracks by holding the 'Alt' key down and dragging the mouse

c. Double click
A user can double click a region of interest to zoom in the tracks. A user can hold the 'Shift' button and double click the tracks to zoom out.

d. Pan
When the tracks are zoomed in, a user can drag anywhere in the track panel to pan.

5. Analyze tracks

The current version of NetGestalt CRC portal provides eleven features to help users analyze the tracks.

a. Network analysis
(i) Module enrichment
For SBT or SCT tracks, NetGestalt CRC portal can help users identify which modules (represented by bars with different length) are significantly correlated with the tracks.

For an SBT, NetGestalt CRC portal uses the Fisher's exact test to identify enriched modules identified from the active network. Enrichment p values are corrected for multiple comparisons by calculating the False Discovery Rates (FDRs).

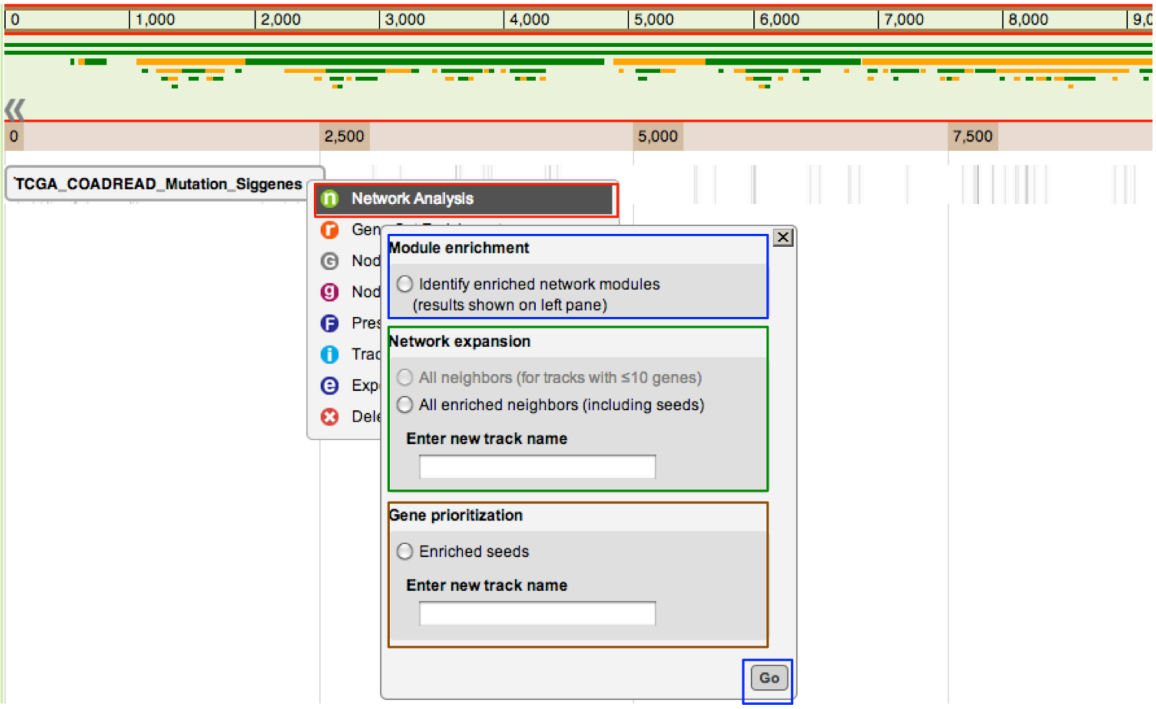

Figure 11. Network analysis in NetGestalt CRC portal

Hovering the mouse over a track name, several buttons for track analysis will be shown in a drop-down menu (Figure 11). Clicking the "Network Analysis" button (red box in Figure 11), several options for network analysis will be shown. Choosing the "Module enrichment" and clicking "Go" (blue box in Figure 11), the enriched network modules will be listed in a table in the "Enrichment Results" section located on the left panel of the page (red box in Figure 12). The user can click column title to sort the results by the corresponding column. The user can navigate through the pages by clicking the buttons right below the table or select the number of entries per page by clicking the drop-down menu at the bottom of the section (see purple box in Figure12). Clicking one entry in the table will add overlapping genes between the enriched module and the original binary track as a new binary track (see green box in Figure12) named by the user. The user can also add all enriched modules in a new composite track by clicking the "Add all related modules" link at the top of the table (see blue box in Figure12). In this composite track, each row represents a hierarchical level of the network; enriched modules are colored in light red and genes in the original binary track are colored in red. The enrichment section can be hidden/shown by toggling the "hide"/ "show" button (see brown box in Figure12).

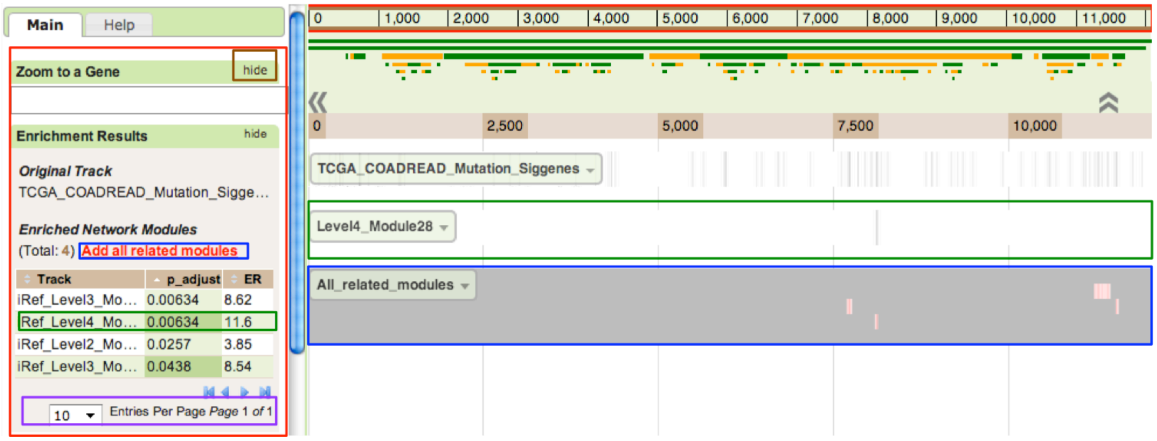

Figure 12. Output for network analysis in NetGestalt CRC portal

For an SCT track, NetGestalt CRC portal uses the Kolmogorov-Smirnov test (KS test) to identify enriched modules. When clicking an entry in the table (or "Add all related modules"), NetGestalt CRC portal will add the leading edge genes in the enriched module as a new binary track (see (Subramanian, et al., 2005) for the definition of leading edge genes). Clicking the "Add all related modules" link at the top of the table will add a new composite track, in which each row represents a hierarchical level of the network; enriched modules are colored in light red and the leading edge genes are colored in red.

(ii) Network expansion

To expand genes in an SBT (*i.e.*, the seed genes) to include other related genes in the network, two options are available in the Section "Network expansion" under "Network Analysis" (green box in Figure 11).

The "all neighbors" option works for SBTs containing 10 or fewer genes, and all direct neighbors of these genes in the network can be retrieved and shown in a new SBT together with the seed genes in the original SBT.

The "enriched neighbors" option works for any SBTs. Specifically, for each non-seed gene in the network, all direct neighbors of the gene are retrieved and evaluated for the enrichment of the seed genes using the Fisher's exact test. All non-seed genes significantly enriched with seed neighbors according to a user defined FDR are identified and shown in a new SBT together with the seed genes in the original SBT.

(iii) Gene prioritization

To prioritize genes in an SBT, NetGestalt CRC portal provides a "Gene prioritization" feature under "Network Analysis" (brown box in Figure 11). Specifically, for each seed gene in the selected SBT, all direct neighbors of the gene are retrieved and evaluated for the enrichment of other seed genes using the Fisher's exact test. Seed genes significantly enriched with other seed neighbors according to a user defined FDR are identified and shown in a new SBT.

b. Gene Set Enrichment

NetGestalt CRC portal compiles information from Gene Ontology (GO) and other five pathway databases including cell map, human cyc, kegg, nci-pid, and reactome to help users identify GO terms or pathways that are significantly correlated with an SCT or SBT track. Figure 13 shows an enrichment result for a binary track based on GO biological process (BP) database. Enrichment analyses are based on the Fisher's exact test and the KS test for SBTs and SCTs, respectively.

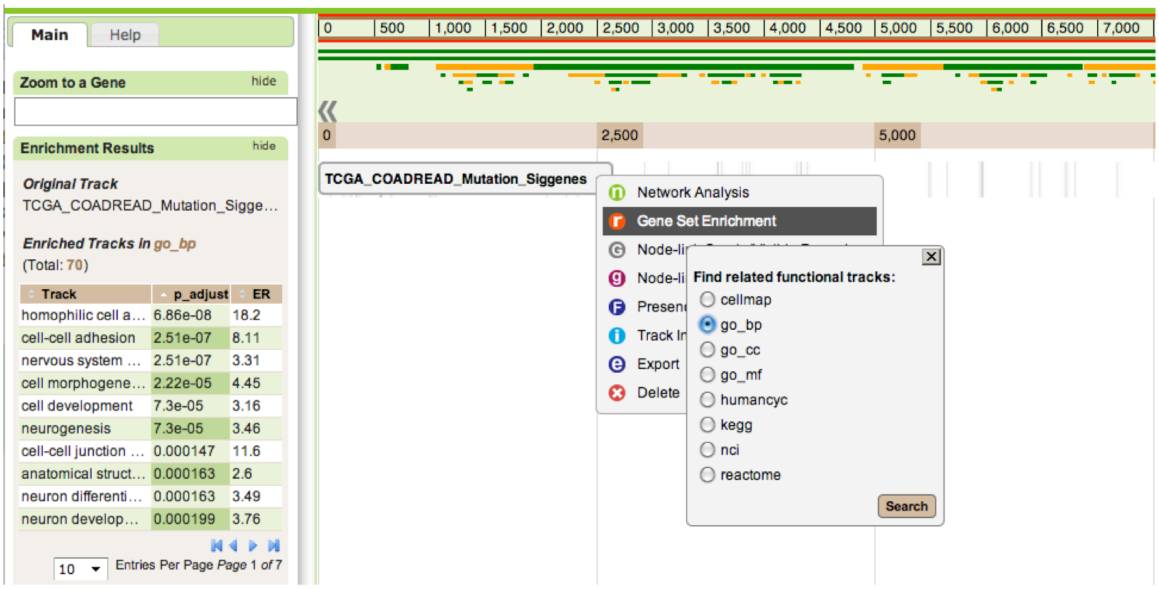

Figure 13. Gene set enrichment analysis in NetGestalt CRC portal

c. Data transformation

To better visualize the CCTs, NetGestalt CRC portal provides a "Data Transform" feature, which allows users to perform gene-wise standardization by subtracting the gene-wise mean or median and set floor and ceiling values for the data (Figure 14). Similarly, using the "Data Transform" feature, users can also set floor and ceiling values for an SCT to improve data visualization.

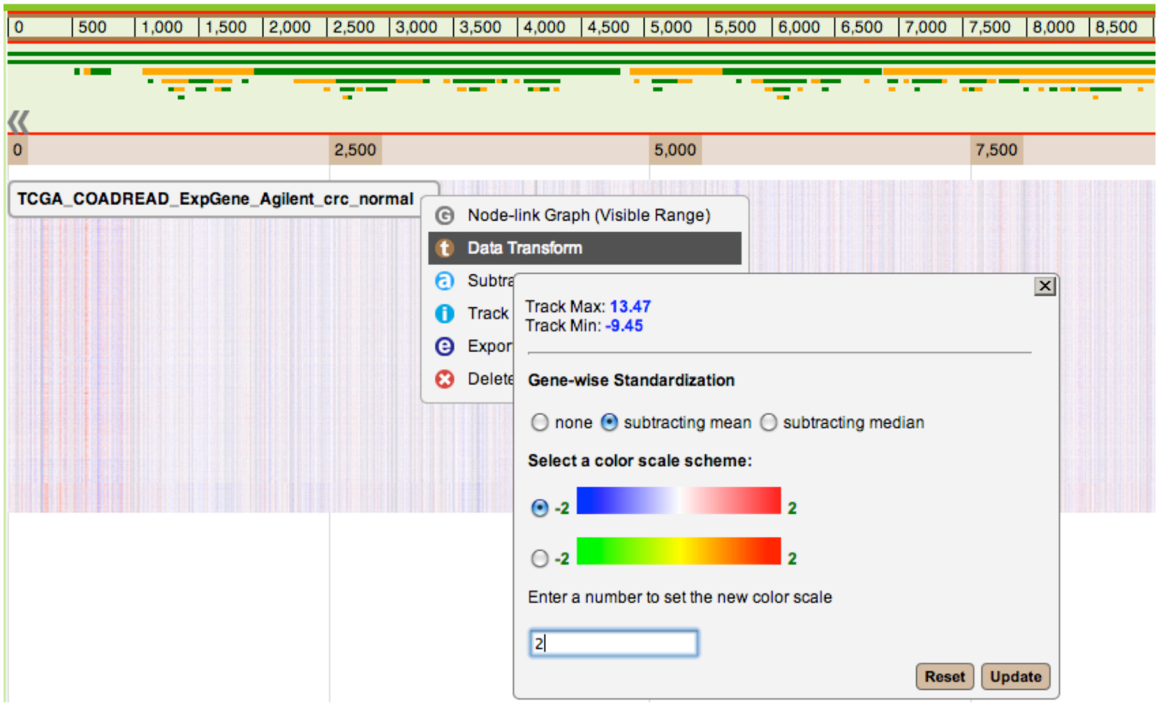

Figure 14. Data transformation in NetGestalt CRC portal

d. Subtrack annotation

For composite tracks (CCT and CBT) containing multiple samples (*i.e.*, subtracks), using the "Subtrack Annotation" feature in the drop-down menu accessible from the track name, users can visualize sample information as a sample heat map with black to green colors for binary data (*e.g.*, Tumor or Normal), categorical data (*e.g.*, Location) and continuous data (*e.g.*, Size) (Figure 15). The order of the sample features can be rearranged, and the samples will be sorted according to the rightmost feature.

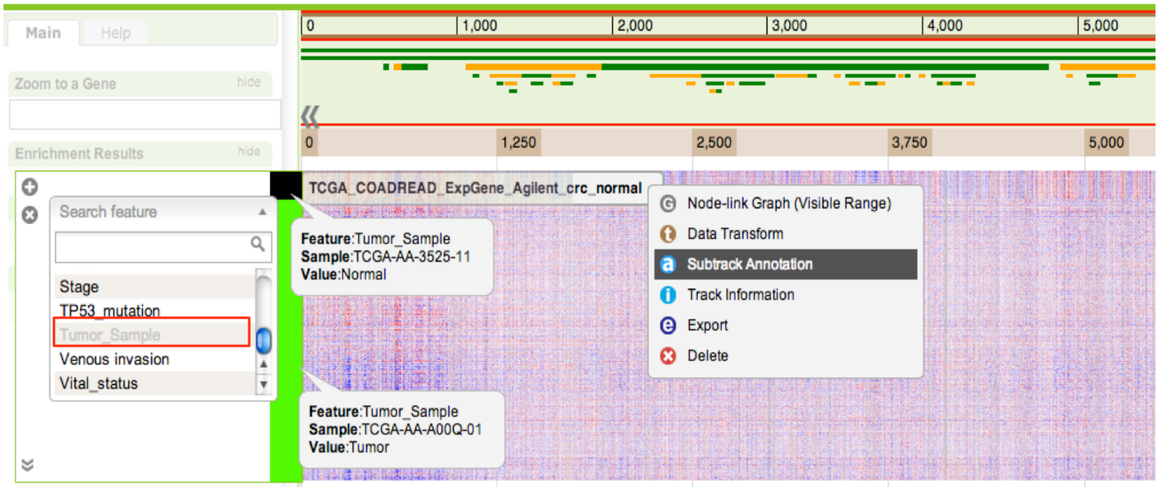

Figure 15 Subtrack annotations in NetGestalt CRC portal

e. Value-based filtering

The "Value-based Filtering" features allows users to filter for interesting genes (*e.g.*, differentially expressed genes) from an SCT. After clicking the "Value-based Filtering" button in the menu (Figure 16), a filtering dialog will be displayed. The maximum and minimum values of the track are shown at the top of the dialog. The user can input the parameters to filter the track. After providing the name of the new track and clicking the "Add Track" button, an SBT will be added to the track viewing area (red box in Figure 16). Genes in the new SBT barplot are colored according to the original values in the SCT, with blue for negative values and red for positive values.

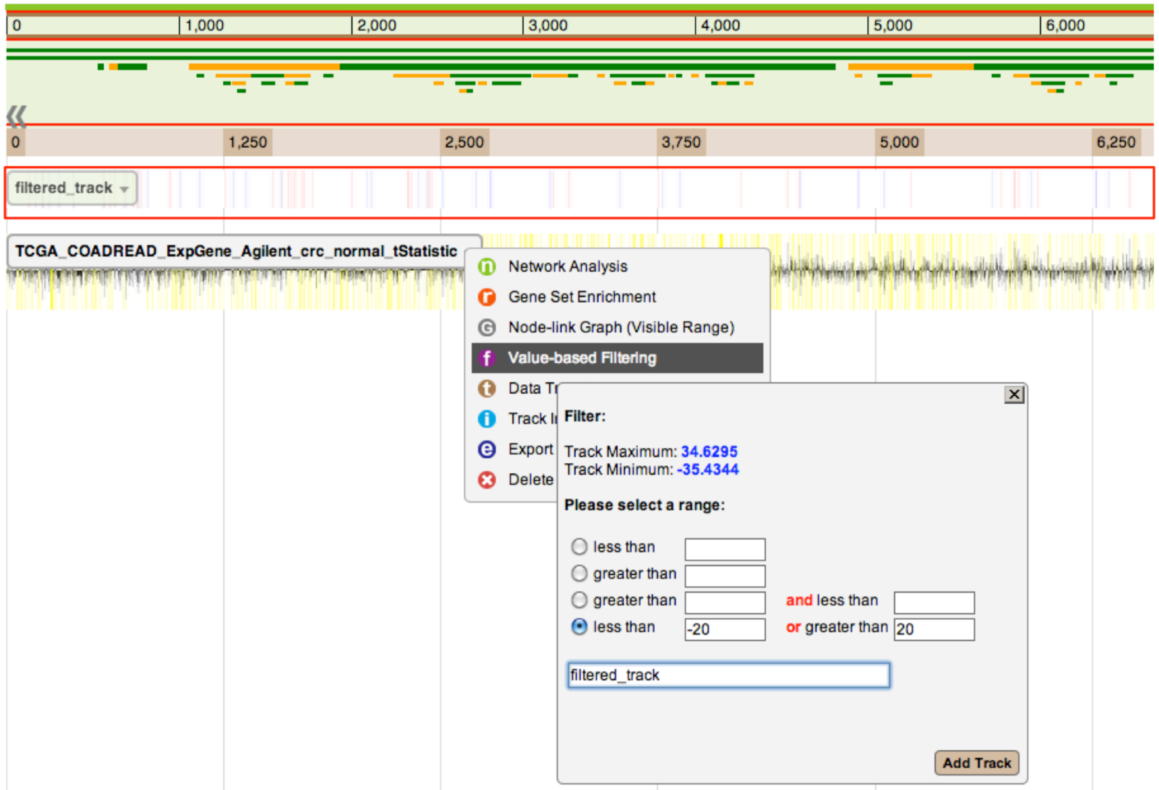

Figure 16. Value-based filtering in NetGestalt CRC portal

f. Presence-based filtering

For SBTs, users can use the "Presence-based filtering" feature to focus on genes present in the current visible range. By clicking the "Presence-based Filtering" button (Figure 17), all genes present in the current visible range from the SBT will be identified, and data for these genes from all tracks in the track viewing area will be displayed in a new webpage (Figure 18). This feature is only activated when the number of genes in the current visible range from the SBT is between 2 to 100.

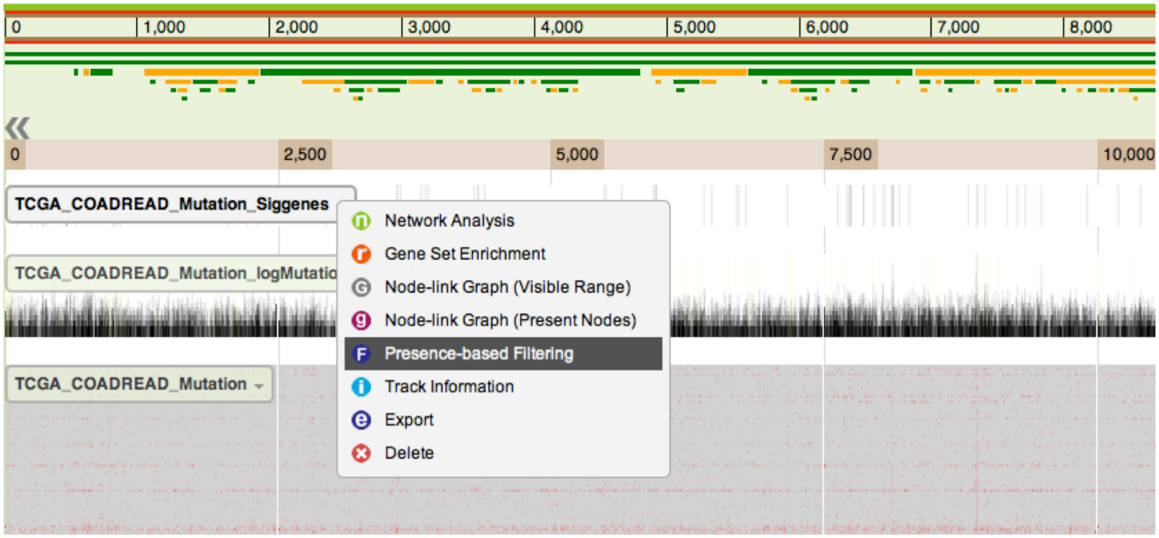

Figure 17. Presence-based filtering in NetGestalt CRC portal

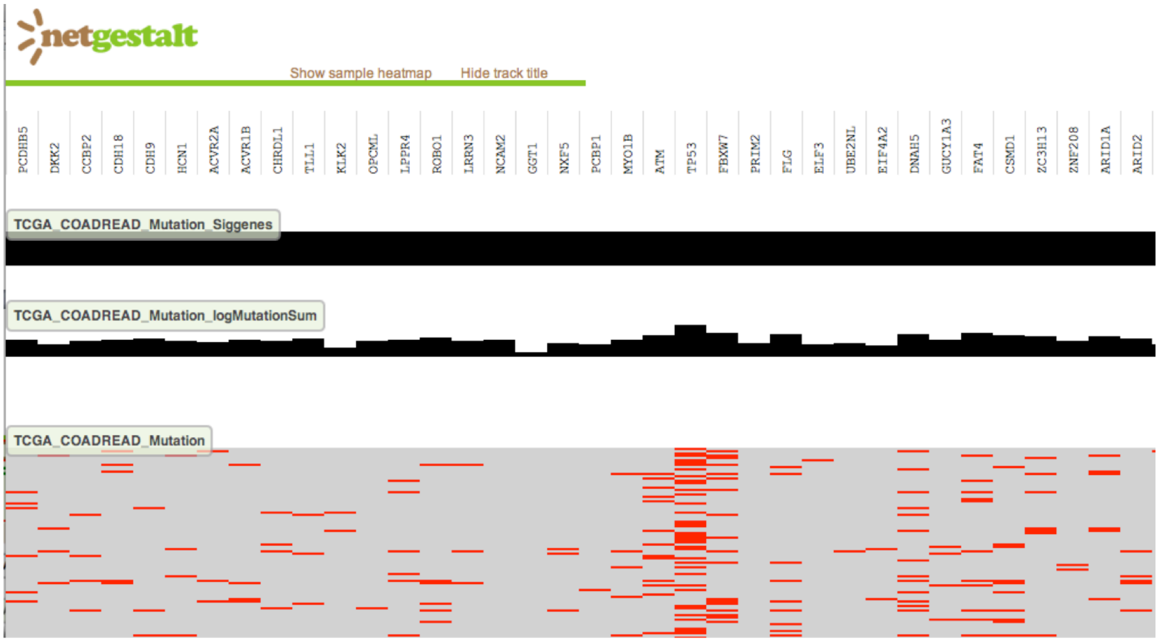

Figure 18. Output for presence-based filtering in NetGestalt CRC portal

g. Node-link Graph

NetGestalt CRC portal also provides the traditional 2D network visualization using the Cytoscape Web plug in.

For CCTs and SCTs, when the number of genes in the current visible range is less than 500, the “Node-link Graph (Visible Range)” button in the drop-down menu will be activated (red box in the top plot of Figure 19). By clicking the “Node-link Graph (Visible Range)” button, network structure for all nodes in the current visible range will

be displayed (left-bottom plot in Figure 19).

For an SCT, NetGestalt CRC portal can color the genes in the network according to their original values in the SCT (right-bottom plot in Figure 19).

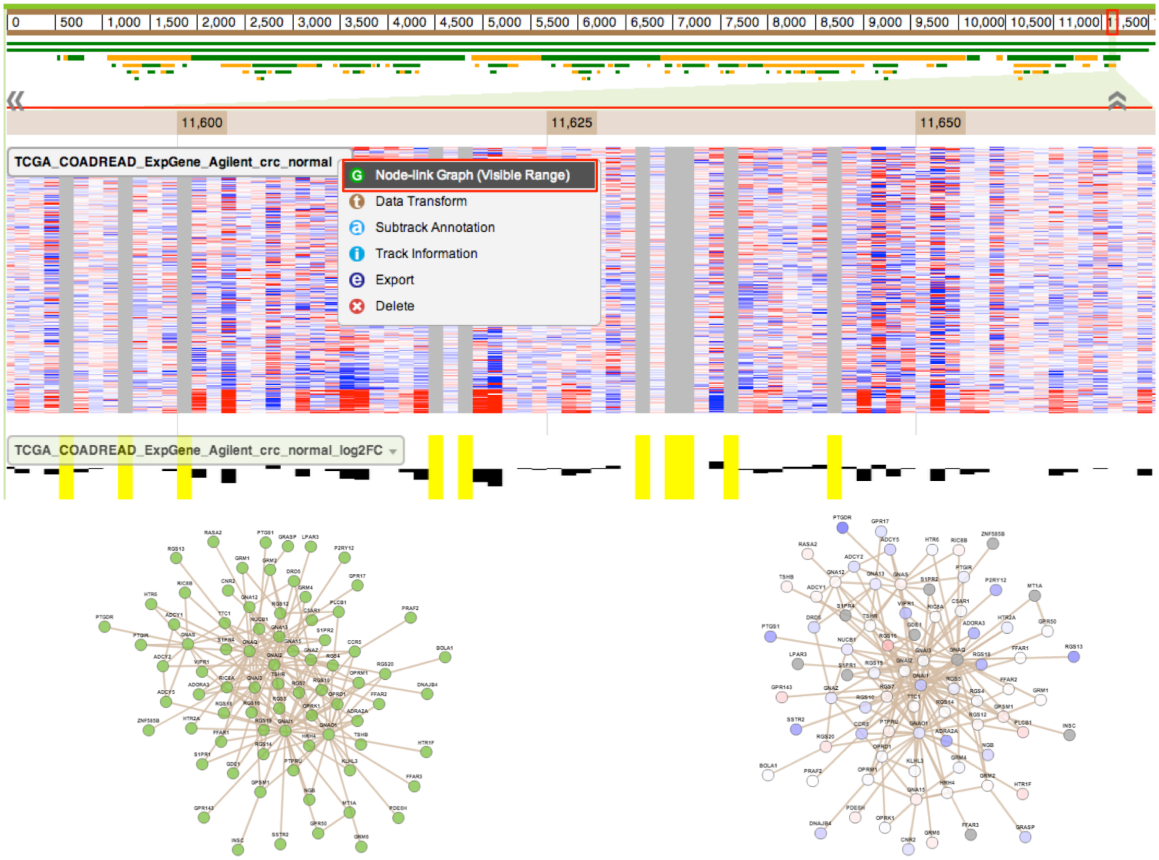

Figure 19. Visualize network structure for CCT and SCT tracks in NetGestalt CRC portal

For SBTs, if genes in the track are colored, the corresponding genes in the network graph will also be colored with the same colors (left-bottom plot in Figure 20). In addition, by clicking the “Node-link Graph (Present Nodes)” button (red box in Figure 20), a network that only contains genes within the visible range and present in the SBT will be displayed (right-bottom plot in Figure 20).

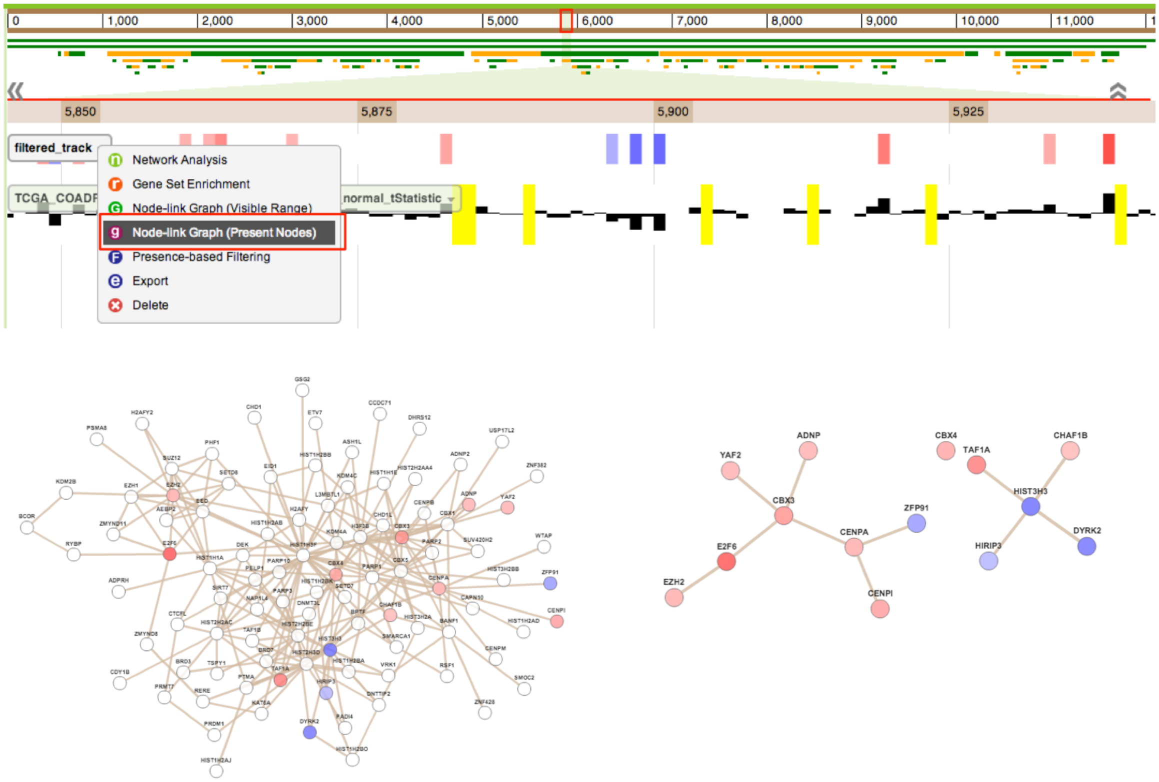

Figure 20. Visualize network structure for SBT track in NetGestalt CRC portal

h. Zoom to a gene

The users can use the "Zoom to a gene" feature to zoom directly to a gene. When typing the gene symbol in the "Zoom to a gene" box (see red box in Figure 21), NetGestalt CRC portal will list all matching symbols below the box on the fly. After selecting a symbol, NetGestalt CRC portal will zoom in to a small region containing the selected symbol and use a vertical red line to highlight the gene in all tracks in the track viewing area (Figure 21).

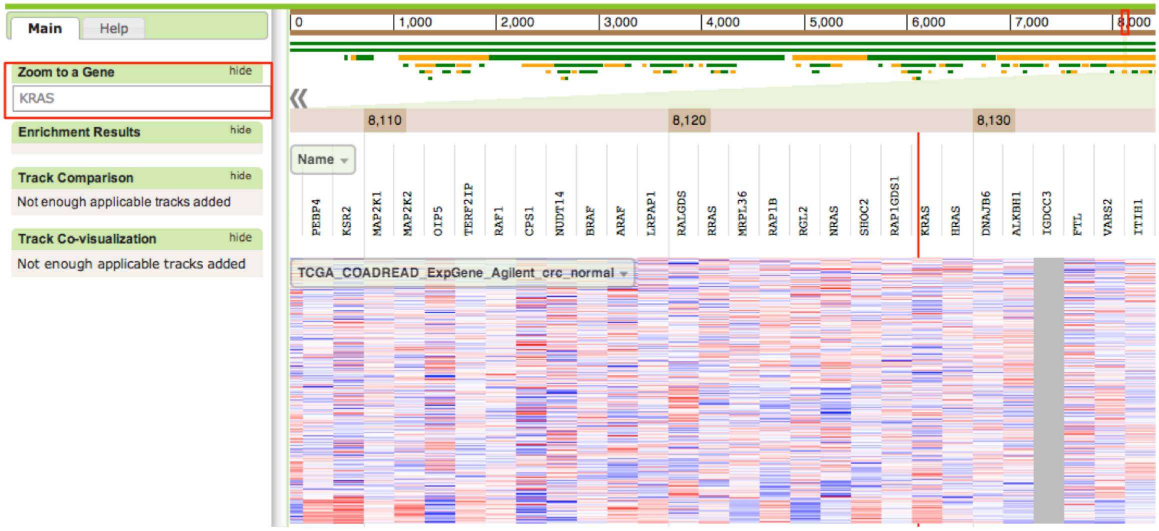

Figure 21. Zoom to a gene in NetGestalt CRC portal

i. Track comparison

NetGestalt CRC portal uses an interactive Venn diagram to help users compare different SBTs. The user can first select the binary tracks from the "Track Comparison" section located in the left panel of the page (see brown box in Figure 22). An SBT track automatically appears in this section when it is added to the track viewing area and will be removed from the section after it is deleted. At most three tracks can be compared at the same time. As soon as tracks are selected, a clickable Venn diagram will be shown below the track names (Figure 22). To help users distinguish different binary tracks easily, NetGestalt CRC portal uses the same color for the selected track name in the "Track Comparison" section, circle in the Venn diagram and upper and lower borders of the binary track visualized in the viewing area. Clicking each part of the Venn diagram will add a new binary track to the track viewing area. For example, the user can click the overlapping part of the Venn diagram and add a new binary track. User can also click the blank region of the Venn diagram to add the union of the genes. Genes in the new binary track are colored corresponding to the colors in Venn diagram.

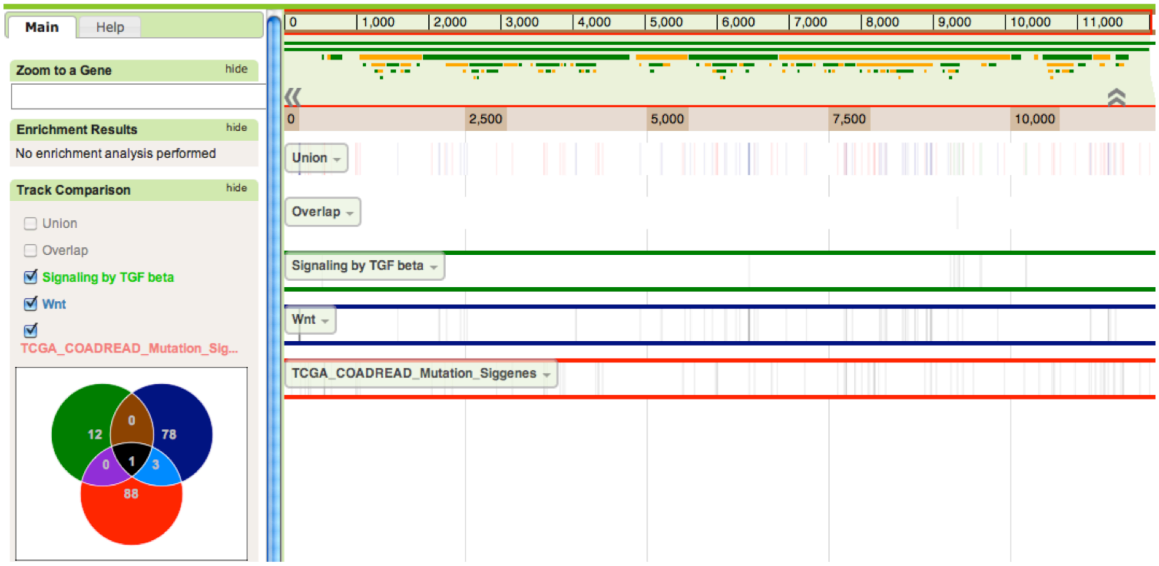

Figure 22. Track comparison in NetGestalt CRC portal

j. Track co-visualization

NetGestalt CRC portal allows users to co-visualize two single tracks (SBT or SCT) in a node-link graph, using the border and fill colors of the nodes to represent data in the two tracks, respectively. First, the user should select the tracks to be co-visualized (SBT or SCT) and the node attributes (border or fill colors) associated with each track in the "Track Co-visualization" section located in the left panel of the page (brown box in Figure 23). After clicking the "G" or "g" buttons, a node-link graph, which contains edges between all genes in the visible range or all present genes in the visible range, will be displayed (left-bottom and right-bottom plots in Figure 23).

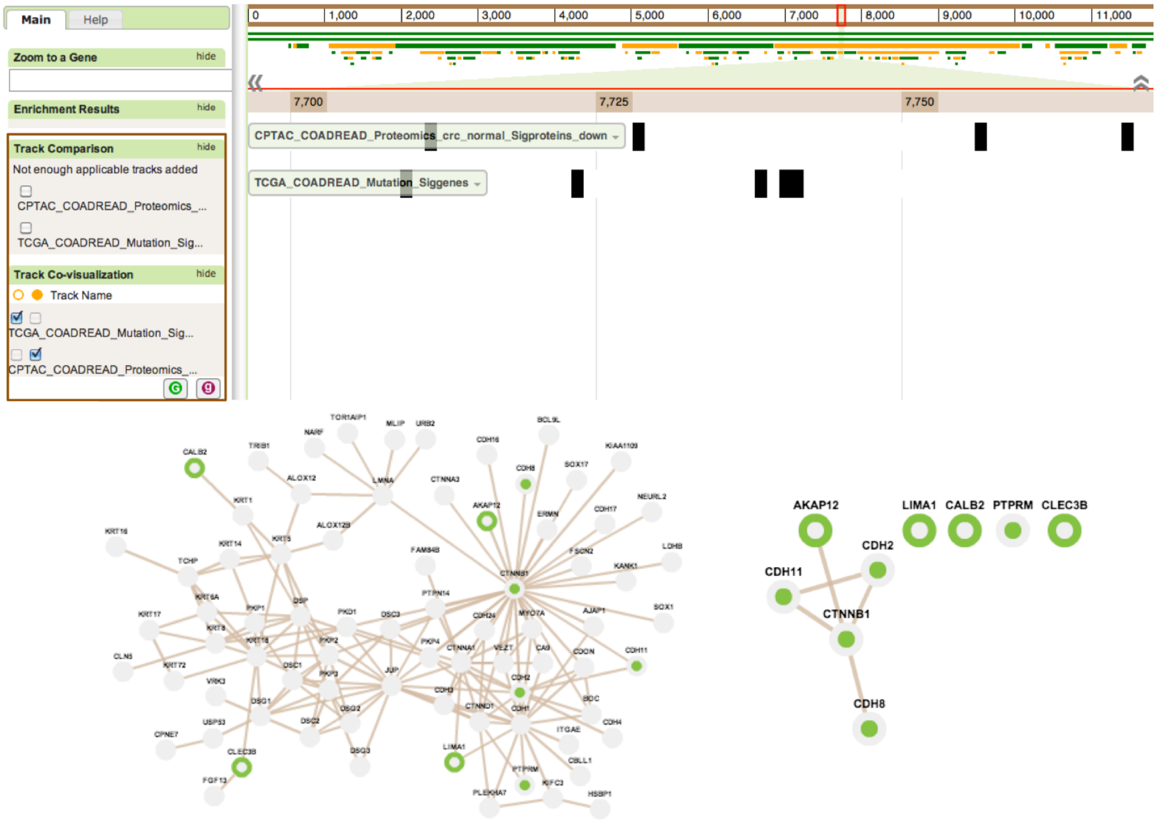

Figure 23. Track co-visualization in NetGestalt CRC portal

k. Switch between different views

The users can visualize the same set of tracks in different network views by making changes using the "Views" → "Select" menu as shown in the Figure 1. This feature allows users to explore the same data sets in the different biological contexts.

6. Data tracks

As shown in Figure 24, the current version of NetGestalt CRC portal contains: (1) genomic, epigenomic, transcriptomic, proteomic, and clinical data for The Cancer Genome Atlas (TCGA) CRC cohort; (2) mRNA expression data from several Gene Expression Ombibus (GEO) CRC cohorts that come with survival information; (3) data from CRC cell lines including drug response, genomic, and transcriptomic data from the Cancer Cell Line Encyclopedia (CCLE) project and shRNA screen data from the Achilles project; and (4) functional tracks including Cell Map pathways, GO Biological Processes, GO Cellular Components and GO Molecular Functions, HumanCyc pathways, KEGG pathways, NCI pathways and Reactome pathways.

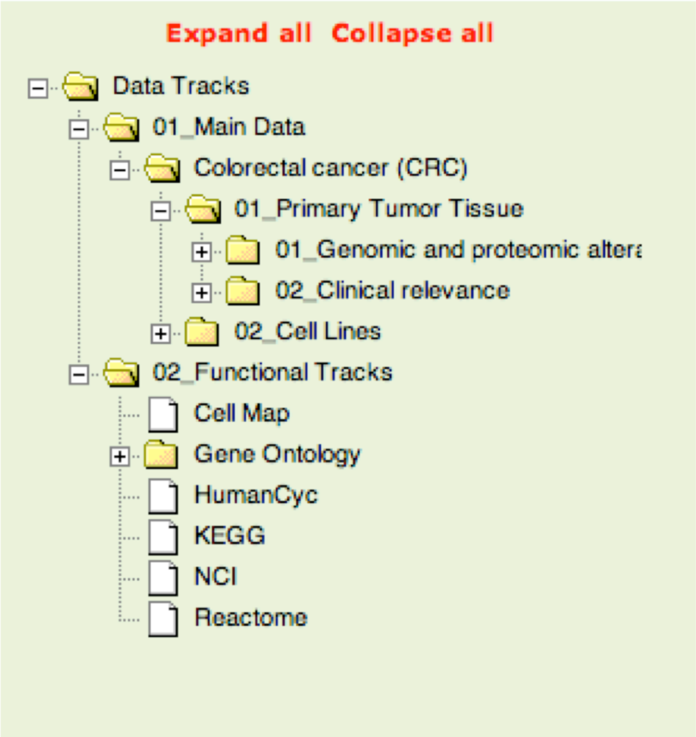

Figure 24 Data tracks in NetGestalt CRC portal

a. Genomic and proteomic alterations in the TCGA CRC tumor cohort

Tracks derived from the multidimensional omics data on the TCGA CRC tumor cohort are included in the Category "01_Genomic and proteomic alterations" (red box in Figure 25). These tracks are divided into nine sub-categories: 01_Omics snapshot, 02_Somatic mutation, 03_Somatic copy number alteration (SCNA), 04_Epigenetic silencing, 05_Differential mRNA expression, 06_Differential protein expression, 07_Correlation between mRNA and protein, 08_Correlation between SCNA and mRNA and 09_Correlation between SCNA and protein.

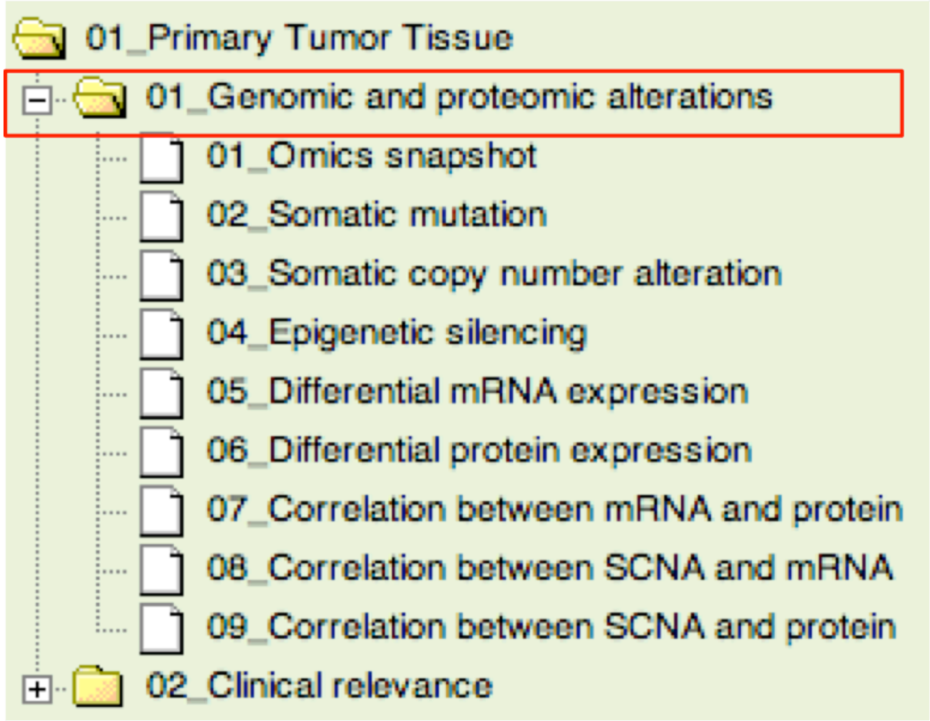

Figure 25 Genomic and proteomic alteration based on CRC tumor tissue

Sub-category 01_Omics snapshot only contains one track "Omics snapshot", which shows the summary of alterations (somatic mutation, somatic copy number alterations, epigenetic alterations, differential expression at mRNA level and protein level) for all the genes based on data from the TCGA CRC tumor cohort.

Sub-category 02_Somatic mutation contains four tracks, including one CBT file recording the binary mutation matrix, two SBT files recording the significantly mutated genes and genes mutated in at least 5% of all the CRC samples and one SCT file recording mutation counts in log scale, i.e. log2(mutation count+1).

Sub-category 03_Somatic copy number alteration (SCNA) contains four tracks, including two CCT files recording the gene level SCNA matrix and focal SCNA matrix, two SBT files recording genes in the focal amplification regions and focal deletion regions.

Sub-category 04_Epigenetic silencing contains two tracks, including one CCT file recording the methylation matrix and one SBT file recording the candidate epigenetically silenced genes.

Sub-category 05_Differential mRNA expression contains six tracks, including one CCT file recording the gene expression matrix, three SCT files recording the t-statistic values, signed -logP (p values were calculated based on t-test) and log2 fold changes and two SBT files recording the up-regulated and down-regulated differentially expressed genes.

Sub-category 06_Differential protein expression contains six tracks, including one CCT file recording the protein expression matrix, three SCT files recording the W-statistic

values, signed -logP (p values were calculated based on Wilcoxon test) and log2 fold changes and two SBT files recording the up-regulated and down-regulated differentially expressed proteins.

For 07_Correlation between mRNA and protein, 08_Correlation between SCNA and mRNA and 09_Correlation between SCNA and protein, each of them contains two tracks, both of which are SCT files. These SCT files record the Spearman's correlation coefficient and corresponding signed -logp.

b. Clinical relevance based on CRC tumor tissue

All the clinical relevant data tracks are included in the Category "02_Clinical relevance" (red box in Figure 26). These tracks are divided into four categories: 01_Clinical relevance snapshot, 02_Survival (including Markers for overall survival and Markers for disease free survival), 03_Stage IV vs Stage I (including Signature genes for Stage IV vs Stage I and Signature proteins for Stage IV vs Stage I), 04_MSI vs MSS (including Signature genes for MSI vs MSS and Signature proteins for MSI vs MSS).

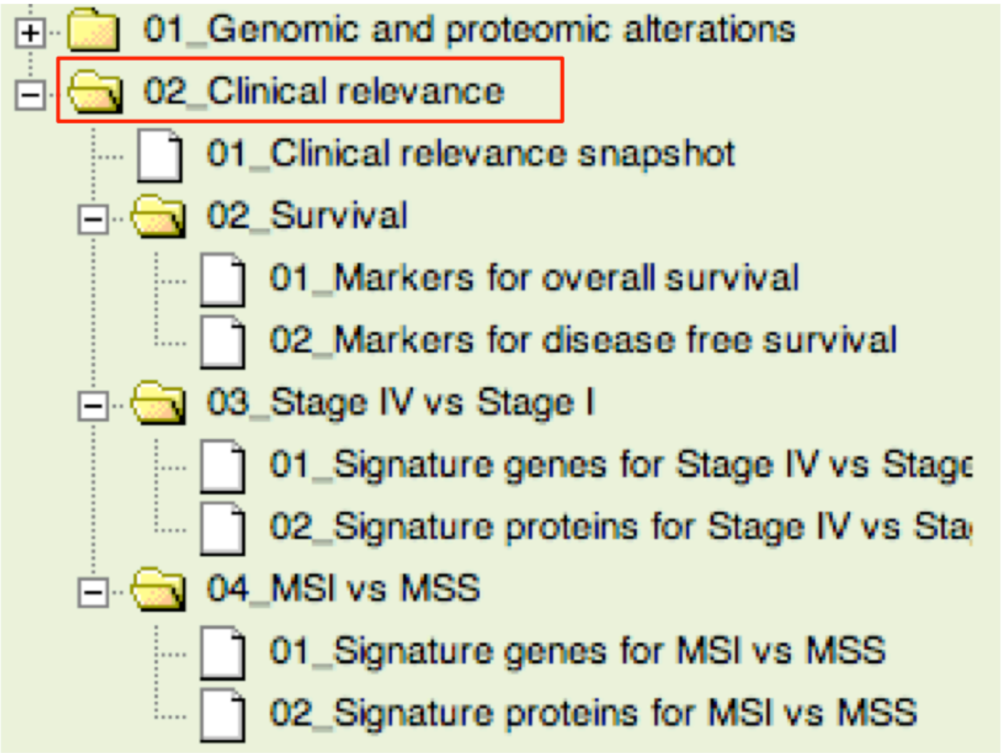

Figure 26 Clinical relevance based on CRC tumor tissue

Sub-category 01_Clinical relevance snapshot only contains one track "Clinical_relevance_snapshot", which shows the summary of clinical relevance including markers for disease free survival, markers for overall survival, signature genes for MSI vs

MSS and signature genes for stage IV vs stage I for all the genes based on multiple datasets.

Sub-category 02_Survival contains two lower level sub-categories 01_Markers for overall survival and 02_Markers for disease free survival. Each of them contains eleven tracks, including (1) eight SCT files recording the correlation (signed -logp and log2(Hazard Ratio)) between gene expression and survival (overall survival or disease free survival) based on Cox regression model and four datasets (two tracks for each of the four datasets); (2) three summary tracks (one continuous track and two binary tracks) summarizing the results based on the four datasets by order statistics (Wang, et al., 2013).

Sub-category 03_Stage IV vs Stage I contains two lower level sub-categories 01_Signature genes for Stage IV vs Stage I and 02_Signature proteins for Stage IV vs Stage I. 01_Signature genes for Stage IV vs Stage I contains fifteen tracks, including (1) twelve SCT files recording the differential expression (t-statistic, signed -logp and log2(Fold Change)) of genes comparing Stage IV with Stage I CRC samples based on t test and four datasets (three tracks for each of the four datasets); (2) three summary tracks (one continuous track and two binary tracks) summarizing the results based on the four datasets by order statistics. 02_Signature proteins for Stage IV vs Stage I contains only three tracks recording the differential expression (W-Statistic, signed -logp and log2(Fold Change)) of proteins comparing Stage IV with Stage I CRC samples based on Wilcoxon test and protein data from (Zhang, et al., 2014).

Sub-category 04_MSI vs MSS contains two lower level sub-categories 01_Signature genes for MSI vs MSS and 02_Signature proteins for MSI vs MSS. 01_Signature genes for MSI vs MSS contains fifteen tracks, including (1) twelve SCT files recording the differential expression (t-statistic, signed -logp and log2(Fold Change)) of genes comparing MSI with MSS CRC samples based on t test and four datasets (three tracks for each of the four datasets); (2) three summary tracks (one continuous track and two binary tracks) summarizing the results based on the four datasets by order statistics. 02_Signature proteins for MSI vs MSS contains only three tracks recording the differential expression (W-statistic, signed -logp and log2(Fold Change)) of proteins comparing MSI with MSS CRC samples based on Wilcoxon test and protein data from (Zhang, et al., 2014).

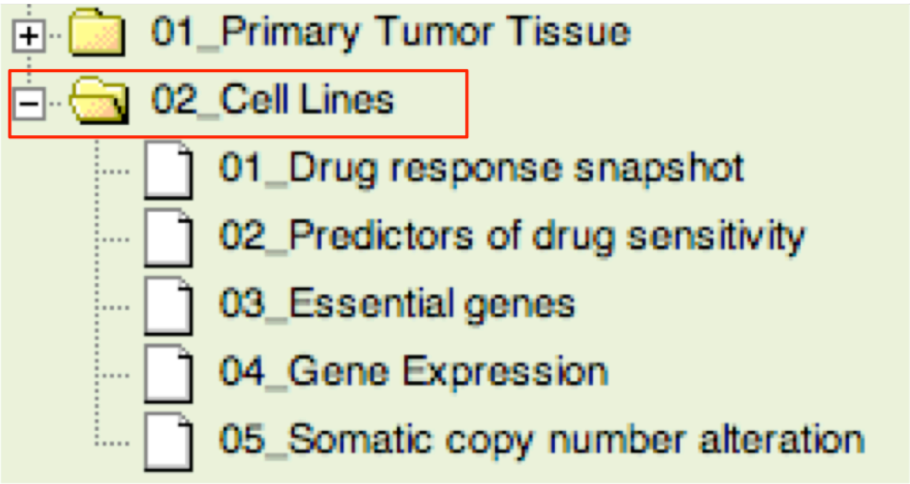

Figure 27 Tracks based on CRC cell lines

c. Tracks based on CRC cell lines

All the data tracks derived from CRC cell lines are included in the Category "02_Cell Lines" (red box in Figure 27). These tracks are divided into five categories: 01_Drug response snapshot, 02_Predictors of drug sensitivity, 03_Essential genes, 04_Gene Expression and 05_Somatic copy number alteration.

Category 01_Drug response snapshot only contains one track "Drug_response_snapshot", which shows the Spearman's correlation coefficient between response (activity area) of the 24 compounds and mRNA expression of all the genes.

Category 02_Predictors of drug sensitivity contains 24 tracks recording the Spearman's correlation coefficient between response (activity area) of 24 compounds and mRNA expression of all the genes separately.

Category 03_Essential genes contains one track "Achilles_RNAi_CRC_Cellline_specific_Essential_Genes", which shows the CRC cell line specific essential genes from Project Achilles (Cheung, et al., 2011).

Category 04_Gene Expression contains one track "CCLE_CRC_ExpGene_AffyU133_2", which shows the gene expression matrix of CRC cell lines from Cancer Cell Line Encyclopedia study.

Category 05_Somatic copy number alteration contains one track "CCLE_CRC_CNA_AffySNP6", which shows the copy number alteration matrix of CRC cell lines from Cancer Cell Line Encyclopedia study.

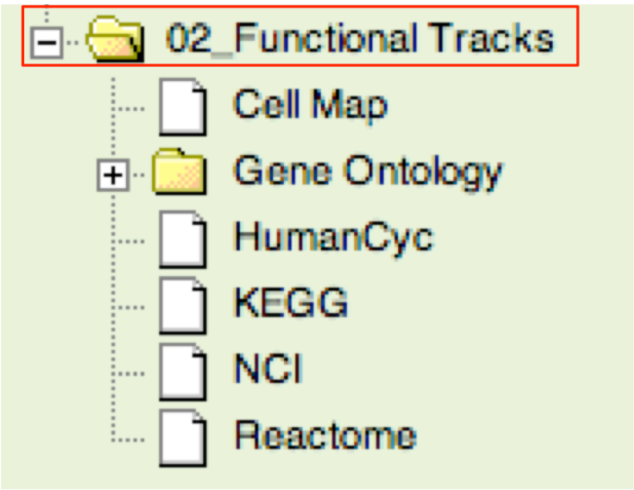

Figure 28 Functional tracks

d. Functional tracks

All the functional tracks are included in the Category "02_Functional Tracks" (red box in Figure 28). These tracks are from six databases: Cancer Cell Map (10 pathways), Gene Ontology (808 Biological Processes, 231 Cellular Components and 383 Molecular Functions), HumanCyc (267 pathways), KEGG (200 pathways), NCI (223 pathways) and Reactome (1108 pathways).

Reference:

Cheung, H.W., et al. (2011) Systematic investigation of genetic vulnerabilities across cancer cell lines reveals lineage-specific dependencies in ovarian cancer, Proceedings of the National Academy of Sciences of the United States of America, 108, 12372-12377.

Shi, Z., Wang, J. and Zhang, B. (2013) NetGestalt: integrating multidimensional omics data over biological networks, Nat Methods, 10, 597-598.

Subramanian, A., et al. (2005) Gene set enrichment analysis: a knowledge-based approach for interpreting genome-wide expression profiles, Proceedings of the National Academy of Sciences of the United States of America, 102, 15545-15550.

Wang, J., et al. (2013) Integrative genomics analysis identifies candidate drivers at 3q26-29 amplicon in squamous cell carcinoma of the lung, Clinical cancer research: an official journal of the American Association for Cancer Research, 19, 5580-5590.

Zhang, B., et al. (2014) Proteogenomic characterization of human colon and rectal cancer, Nature, (In press).
